# Shared cultural ancestry predicts the global diffusion of democracy

**DOI:** 10.1017/ehs.2022.40

**Published:** 2022-09-19

**Authors:** Thanos Kyritsis, Luke J. Matthews, David Welch, Quentin D. Atkinson

**Affiliations:** 1 School of Psychology, University of Auckland, New Zealand; 2 RAND Corporation, Boston, Massachusetts, USA; 3 Faculty, Pardee RAND Graduate School, Santa Monica, California, USA; 4 Centre for Computational Evolution, University of Auckland, New Zealand; 5 School of Computer Science, University of Auckland, Auckland, New Zealand

**Keywords:** democracy, cultural diffusion, cultural evolution, language, religion

## Abstract

Understanding global variation in democratic outcomes is critical to efforts to promote and sustain democracy today. Here, we use data on the democratic status of 221 modern and historical nations stretching back up to 200 years to show that, particularly over the last 50 years, nations with shared linguistic and, more recently, religious ancestry have more similar democratic outcomes. We also find evidence that for most of the last 50 years the democratic trajectory of a nation can be predicted by the democratic status of its linguistic and, less clearly, religious relatives, years and even decades earlier. These results are broadly consistent across three democracy indicators (Polity 5, Vanhanen's Index of Democracy, and Freedom in the World) and are not explained by geographical proximity or current shared language or religion. Our findings suggest that deep cultural ancestry remains an important force shaping the fortunes of modern nations, at least in part because democratic norms, institutions, and the factors that support them are more likely to diffuse between close cultural relatives.

## Graphical abstract

**Figure:**
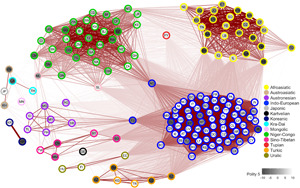

**Social media summary:** Countries with common linguistic and religious ancestry are more similar across a range of democratic outcome measures.

## Introduction

**Figure 1.**
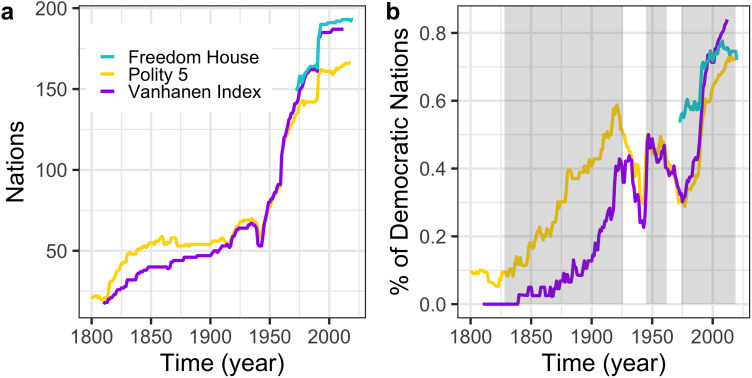
Democracy measures through time. (a) Number of nations sampled through time for three democracy datasets, Freedom House (cyan), Polity 5 (yellow), and the Vanhanen Index (purple). (b) Percentage of democratic regimes across the same three datasets. A nation was counted as democratic if it was classified as ‘free’ or ‘partly free’ by Freedom House, or had a Polity 5 score greater than 0 or a Vanhanen index greater than 5. The three waves of democratisation (Huntington, 1991) are visible across the three datasets (shaded in grey, first wave – 1828–1926; second wave, 1945–1962; third wave, 1974 to present).

Democratic self-determination is viewed today as a fundamental human right (UN General Assembly, 1948), yet most major civilisations throughout human history have been autocratic. Modern national democracies are the product of a series of social and political reforms that began to emerge since the early nineteenth century and spread rapidly across the globe, replacing autocracies in what is sometimes characterised as three waves of democratisation: an initial ‘slow’ wave beginning in the US and culminating in the emergence of several European democracies at the end of the First World War (1828–1926); a second wave linked to the process of decolonisation following the end of the Second World War (1945–1962); and a third wave comprising a succession of transitions in Western Europe, Latin America, the Pacific, Eastern Europe after the fall of communism, and sub-Saharan Africa (1974 to present; Huntington, 1991). Despite the challenge of defining democracy and of quantifying democratic progress (Coppedge et al., 2015), this general pattern is observed across a range of different democracy measures (Figure 1).

There is a wealth of scholarship seeking to explain the rise of modern democracies. Much of this research has focused on internal, country-level predictors of democratic progress, including economic growth (Boix, 2011; Inglehart & Welzel, 2009; Knutsen et al., 2019), technology (Mays & Groshek, 2017), education (Glaeser et al., 2007), human development (Doorenspleet, 2019; Landman, 2013), civic values (Ruck et al., 2020), and happiness (Inglehart, 2009). Alongside this work, others have begun to examine how external connections between nations shape the global diffusion of democracy (Elkink, 2011; Manger & Pickup, 2016; Matthews et al., 2016; O'Loughlin et al., 1998; Rhue & Sundararajan, 2014; Spolaore & Wacziarg, 2018; Wejnert, 2005). Perhaps the simplest such model is one in which the determinants of democracy diffuse spatially as nations adopt the institutional practices of their geographical neighbours. Consistent with this, a number of studies have shown that spatial proximity predicts similar democratic outcomes (Elkink, 2011; O'Loughlin et al., 1998; Wejnert, 2005). More recent work has also explored the effects of international trade connections (Manger & Pickup, 2016; Rhue & Sundararajan, 2014), digital connectedness (Rhue & Sundararajan, 2014), and membership of intergovernmental organisations (Torfason & Ingram, 2010).

However, less work has examined how older cultural connections between nations shape the political landscape today. While some research has attempted to incorporate cultural history in predictive models, this has tended to rely on a limited set of cultural indicator variables (Acemoglu & Robinson, 2012; Inglehart & Welzel, 2005; Lotan et al., 2011), rather than seeking a general framework with which to quantify and control for ancestral cultural connections between nations. The relative paucity of work in this area is surprising for two reasons. First, shared cultural history is widely acknowledged as a potentially important predictor of a range of national outcomes (Huntington, 1991; Nunn, 2012; Spolaore & Wacziarg, 2013). Second, the importance of controlling for statistical non-independence between nations owing to shared cultural history, even when investigating putatively internal drivers of political change (Bromham et al., 2018; Wejnert, 2005), is widely recognised and known as ‘Galton's problem’ (Naroll, 1961).

There are two primary mechanisms by which cultural ancestry can affect modern variation in national outcomes. First, nations can directly inherit norms, values and institutions that are passed vertically down cultural lineages so that close cultural relatives share more such features. For example, the British colonies Canada, Australia and New Zealand inherited and retain elements of the British political and legal system (Oliver, 2005). Second, ancestral cultural distance between nations may create cultural ‘barrier effects’, limiting more recent horizontal diffusion of novel cultural traits (Spolaore & Wacziarg, 2009). Conversely, innovations may be more likely to diffuse horizontally between nations that are close cultural relatives, either because cultural relatives are more likely to build and maintain the kinds of social and economic connections that allow innovations to spread or, even in the absence of greater connectivity between relatives, inherited cultural similarities may still make innovations more likely to be adopted by close relatives. This process can be considered a form of ‘cultural contagion’, akin to the social contagion of behaviour in social networks (e.g. Christakis & Fowler, 2013). The Arab Spring, for example, spread among neighbouring countries with close linguistic and religious ties (Howard & Hussain, 2013; Lotan et al., 2011; State et al., 2015).

Pioneering work in this area has already linked a range of national outcomes to genetic distance between populations, used as a proxy for shared cultural ancestry (Spolaore & Wacziarg, 2009, 2018). This includes work by Spolaore and Wacziarg (2018) showing that differences in democracy outcomes between nations are predicted by their relative genetic distance to the ‘institutional frontier’ (US). However, genetic diversity is highly correlated with geography (Novembre et al., 2009) and only indirectly related to cultural ancestry (e.g. Posth et al., 2018), making interpretation of any such relationship difficult. An alternative approach is to infer cultural ancestry based on ancestral relationships between the world's languages (Bouckaert et al., 2012, 2018; Gray et al., 2009; Mace & Holden, 2005). These language family trees represent genealogies of cultural ancestry that more reliably track vertical relationships of descent between populations than do genes (Pagel, 2009). Language trees have been shown to powerfully predict variation among traditional societies across a range of traits, including descent systems (Opie et al., 2014), political complexity (Currie et al., 2010; Sheehan et al., 2018) and religion (Watts et al., 2016). However, this approach has not yet been widely applied to predict socio-political outcomes among large modern societies.

In a recent proof-of-concept study, Matthews et al. (2016) showed that language ancestry can indeed predict the diffusion of democracy. Controlling for spatial proximity and a range of other factors, democratic change – as measured by Polity IV scores (Marshall et al., 2002) – among Indo-European-speaking nations was more likely to spread between linguistically similar nations. Subsequent work by Currie et al. (2021) has shown that, again among Indo-European-speaking nations, linguistic distance from the US predicts the timing of the adoption of democracy. Others have also used language ancestry to address Galton's problem when predicting putative internal drivers of political change (Bromham et al., 2018; Ruck et al., 2020; Spolaore & Wacziarg, 2018). However, attempts to quantify the effect of language ancestry on democratic outcomes have either not made use of high-resolution language trees or have been restricted to a single language family (Matthews et al., 2016; Currie et al., 2021). Similarly, despite a long tradition of interest in the role played by religion in shaping national outcomes (McCleary, 2008; Schulz et al., 2019; Weber, 1904; Huntington, 1991), cultural phylogenetic techniques have not been used to investigate analogous effects owing to religious ancestry. Lastly, prior work has tended to focus on a single measure of democracy, but democracy is a multifaceted construct (Coppedge et al., 2015) that includes, among others, electoral participation, democratic freedoms and rights, and the presence of democratic institutions, democratic principles and values, all of which can be measured in various ways (see Coppedge et al., 2015; Skaaning, 2018 for a comparison). Consequently, the precise scope of any cultural ancestry effects on democratic outcomes remains unclear.

**Figure 2.**
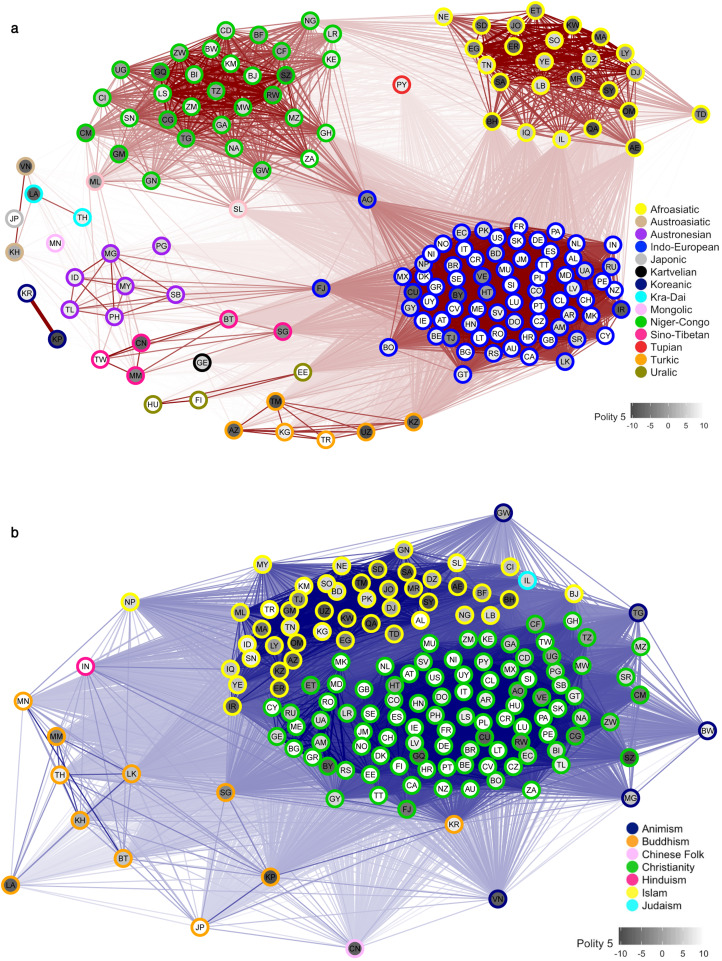
Global variation in democracy across networks representing linguistic and religious connections between nations. (a) Variation in Polity 5 scores for the year 2012 across a global network of linguistic connections (edges) between 163 contemporary nations (nodes). Lighter node hues indicate more democratic nations. Node proximity and edge transparency reflect linguistic connections based on all languages spoken by at least 1 permille of each nation’s population, weighted by their respective percentages (see Methods). Node borders are colour-coded by language family of the nation's majority language (see Table S1 for assignments and ISO codes). (b) As for (a) but showing religious connections based on percentage adherents to 28 major religions. Node borders are colour-coded by the nation's majority religion (see Table S1 for assignments and ISO codes). For a comparison with Freedom House and Vanhanen Index data for the same year, see Figures S1–2.

Here, we extend research on the global diffusion of democracy by quantifying the degree to which three different democratic indicators are independently predicted by two layers of cultural influence – linguistic and religious ancestry (see Figure 2) – over the course of Huntington's (1991) three waves of democracy. First, we test whether nations that are close linguistic or religious relatives are similar in their democratic outcomes at any given point in time. Since linguistic and religious ancestry are themselves geographically clustered, we control for geographical proximity, allowing us to determine whether the predictive power of cultural ancestry goes beyond what would be expected by a simple model of geographical diffusion across the landscape. Second, we examine the role of cultural contagion by testing whether, controlling for a nation’s current democratic status, its future democratic status is predicted by the democratic status of its cultural relatives and geographical neighbours.

To answer these questions, we constructed global networks of linguistic and religious affiliations between all nations based on ancestral relationships between languages and religions together with the number of speakers/adherents in each nation (see Methods; Ruck et al., 2020). We combined these data with democratic panel data from three different sources using independent codings that stretch back up to 220 years: Polity 5 (Marshall et al., 2019; 1800–2018), which emphasises restraints on executive functions; Vanhanen's Index of Democracy (Vanhanen, 2000; 1810–2012), focusing on nations’ electoral participation and competition; and Freedom House's Freedom in the World dataset (Freedom House, 2021; 1972–2020), measuring individual rights and freedoms. Between them, these datasets cover 221 modern and historical nations over the period 1800–2020 (Figure 1; see Methods), allowing us to trace and model the spread of modern democracy from its birth all the way to the present.

## Methods

### Democracy

We studied three time-series country-level indicators from independent datasets that capture different aspects of democracy. *Polity 5* (Marshall et al., 2019) measures nations’ autocratic or democratic character, emphasising restraints on executive functions, and covers the period 1800–2020. We used the revised combined *polity2* measure, which ranges from −10 (autocracy) to 10 (democracy). *Vanhanen's Index of Democracy* from the Polyarchy dataset (Vanhanen, 2000) measures nations’ electoral participation and competition over the years 1810–2012. The index ranges from 0 (no participation/competition) to 50 (full participation/competition). *Freedom House's Freedom in the World* survey (FH) (Freedom House, 2021) measures individual rights and freedoms in nations over the period 1972–2020. We used the combined reversed score which ranges from 0 (not free) to 12 (free).

Democracy data were aggregated in the statistical software R (R Core Team, 2020) using the package *democracyData* (Márquez, 2017). Data from later years not covered by the package were added manually. Pearson's correlation between Polity and the Vanhanen Index across all years was 0.76, between Polity and FH 0.89, and between Vanhanen and FH 0.82, all significant at a level of *p* < 0.001.

### Country sample

We sampled all years for all UN member states and all historical nations represented in the three democracy datasets. This resulted in a list of 221 modern and historical nations (see Table S1), spanning a period of 220 years across three democracy outcomes and 41,638 observations in total. Data availability and therefore nations sampled by year varied over time, from just 25–50 nations (covering all continents, including Asia, North and South America, Africa, and Europe) at the beginning of the nineteenth century to 160–200 nations in the twenty-first century (see Figure 1; note that the Freedom House data only begin in 1972). This shift in sample size reflects both less source data available on nations earlier in the time series and a real increase in the number of internationally recognised sovereign states around the globe (since the establishment of the UN in 1945, the number of recognised member states has increased from 51 to 193 today, including but not restricted to new sovereign states associated with decolonisation and the fall of communism). Figure 2 shows the global distribution of Polity 5 scores across linguistic and religious networks of 163 contemporary nations (see Figures S1–2 for similar plots of Vanhanen and Freedom House data).

We note that nations that amalgamate or split up are treated as separate entities by the Polity 5, Vanhanen, and Freedom House datasets, and given different country codes. For example, Korea (KOR) ceased to exist in 1945 when South Korea (KR) and North Korea (KP) were established. We used the same system, treating these as three separate nations with their own location and cultural affiliation data. This has no effect on our cross-sectional analyses because we are only interested in democratic similarity between nations that existed in any given year. In the longitudinal analyses, since we predict a nation's democracy score at Time 2 (T2) from the democracy score of its geographical and cultural neighbours at Time 1 (T1), controlling for its own democracy at T1, we only track democratic change in countries that were in existence at T1 and T2, and the effects we describe therefore relate to internal change within established nations, rather than transitions associated with the amalgamation or the splitting of nations.

### Cultural connections

We quantified two measures of cultural connections between nations in our sample, linguistic and religious (see Figure 2), based on the methodology outlined in Eff (2008). First, we used known ancestral relationships between all languages and between all religions to calculate pairwise cultural similarity scores between them (see the next sections for details on these calculations). We then used these scores to derive pairwise linguistic and religious connection measures between all nations. Our linguistic connection measure considered the cultural similarity of all languages spoken by at least 1 permille of the population in any pair of nations, weighted by respective speaker percentages in each nation, as recorded in Ethnologue 21 (Eberhard et al., 2018). We repeated the same process for religion, by comparing the ancestral relationships of 28 major religions across all nations (Figure S3), weighted by respective adherent percentages from National Profiles (Finke & Grim, 2019) of the Association of Religion Data Archives (ARDA; Brown et al., 2018). We ignored ARDA's ‘other’ and ‘syncretic’ religions, as well as ‘neoreligionism’ and ‘atheism’, because they represent broad categories that do not necessarily imply common ancestry. For historical states we made approximate estimates for percentages based on the CIA factbook (Central Intelligence Agency, 2018) archives as well as other sources (Bjørklund, 1970; Izady, 2018; Joshua Project, 2018; McNally et al., 1893; Smiley, 1839; Statistics Canada, 2009; Wittman, 1907). The linguistic or religious connection *c_rk_* between two nations *r* and *k* was thus defined as (1):1
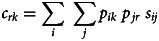
where *p_ik_* is the percentage of the population in nation *k* speaking language *i* (or adhering to religion *i*), *p_jr_* is the percentage of the population in nation *r* speaking language *j* (or adhering to religion *j*) and *s_ij_* is the proximity measure between languages *i* and *j* (or religions *i* and *j*). The following two sections explain how values *s_ij_* were calculated for linguistic and religious data.

### Language ancestry

Quantifying linguistic ties between nations required a measure of genealogical relationships between languages themselves. To this end, we compiled a global language tree of all ~7000 languages based on genealogical assignments specified in Glottolog 3.0 (Hammerström et al., 2018). After removing unattested, unclassified or pidgin languages, as well as dialects, we acquired a single unrooted, unresolved language tree with 7186 languages. To generate branch lengths and produce an ultrametric global language family tree, we used Grafen's method (Grafen, 1989), which assigns each node a height value based on the number of leaves in the subtree minus 1, scales the values so that the root height is 1 and the height of leaves is 0, and raises them to the power ‘rho’ (> 0). The difference in these values between a node and its parent is their branch length. Tree manipulation took place in R using the *compute.brlen* and the *cophenetic.phylo* functions of the R package *ape* (Paradis et al., 2004). This resulted in a 7186 × 7186 matrix of pairwise distances between all languages; values were reversed to proximities and used as *s_ij_* in equation (1) above.

### Religion ancestry

We constructed a family tree showing genealogical relationships between 28 religious lineages (Figure S3), informed by historical sources and key historical events and dates (see Supplementary Materials). To account for evidence of horizontal transmission between some traditions, rather than a single tree, we derived a sample of eight religion trees in which the alternative paths of inheritance represented by the different horizontal transmission events are captured by different bifurcating combinations of splits that alter tip-to-tip distances. The small number of broadly defined religious traditions we considered (28 as compared with ~7000 languages) meant that it was feasible to use historical information to date the age of most of the traditions, and the hybridisation events. While the precise chronology of the tree is contestable, we judged some approximation of time depth preferable to not attempting to include historical information in branch lengths. Moreover, as we show in our robustness checks, using alternative branch lengths that do not include time–depth information produces qualitatively similar results. We extracted the pairwise distance matrices from each tree, and then averaged them to produce a working model for this analysis. Paths that did not involve horizontal transmission were unaffected by this procedure, while paths that crossed a horizontal transmission event are the mean value of the alternative pathways represented by that event. This religion tree can be seen in Figure S3. The resulting 28 × 28 matrix captured pairwise distances between these religions; values were reversed to proximities and used as *s_ij_* in equation (1) above.

### Geographic proximity

In order to control for geographical relations between nations, our analyses included a geographical proximity matrix. We assigned each nation the point location of its capital city, since the population bulk in a nation is typically gathered in and around its capital. We obtained coordinates for capitals of the countries in our sample using the R package *maps* (Deckmyn, 2018) and Google Maps, and calculated pairwise geodesic distances, which accounts for the Earth's ellipsoid shape, using the *distGeo* function of the R package *geosphere* (Hijmans et al., 2017). Following convention, we log-transformed geographical distance by calculating its logarithm in base 10. Values could be reversed to proximity or distance accordingly.

### Cross-sectional analysis

For our cross-sectional analyses we used a dyadic regression approach with random effects (Karimov & Matthews, 2017). This approach differs from standard linear regression in that all predictor and outcome variables represent pairwise (dyadic) differences rather than monadic values. The lower triangles of symmetric distance matrices, i.e. undirected networks where the distance from node A to B is the same as the distance from B to A, can be converted to vectors of pairwise differences that are then regressed using linear regression. Although the end result is a vector, it is still based on matrix data, as values represent distances between data points, rather than independent scores. In order to control for the repetition of cases, the identities of each node in the pair are added as random effects. The dyadic regression with random effects approach was initially developed in the context of longitudinal social network analyses by de Nooy (2011) and O'Malley and Christakis (2011), who also offer mathematical proofs of the validity of the approach. Simulations also indicate that dyadic regression with random effects outperforms alternative autoregressive models for the type of data we consider here (Karimov & Matthews, 2017).

**Figure 3.**
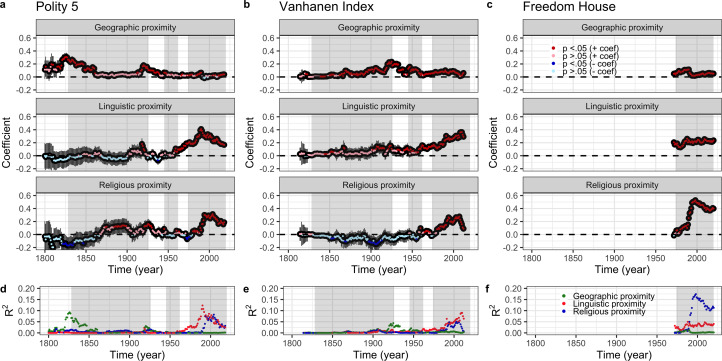
Independent effects of geographical, linguistic and religious connections predicting democracy. Pairwise differences in democracy between nations were simultaneously regressed on geographical, linguistic and religious connections between nations, at each time slice for which data was available, resulting in 466 cross-sectional models. Multiple regression standardised coefficients of the three predictors are presented separately for Polity 5 (a, 1800–2018), the Vanhanen Index (b, 1810–2012) and Freedom House data (c, 1972–2020), with 95% CI annotated. The direction and significance of effects are colour-coded: red for significant positive coefficients (*p* < 0.05), pink for non-significant positive coefficients, dark blue for significant negative coefficients (*p* < 0.05) and light blue for non-significant negative coefficients. (d–f) Semi-partial coefficients of determination (*R*^2^) are displayed below the respective models and outcome variables from (a–c), indicating the proportion of variance in democracy explained by geography (green), language (red) or religion (blue), after controlling for the other two variables. The three waves of democratisation are highlighted in grey on all graphs.

For each of the three democracy indicators, we predicted pairwise differences in democracy between nations from their linguistic, religious and geographical connections. Data for each year for each of the three indicators constituted a separate analysis. First, we converted democracy scores for each year to a matrix of between-country differences in democracy. We also subset our three predictors, pairwise linguistic, religious and geographical connections, to include the same nations as the democracy matrix for each year. We standardised all dependent and independent variables by dividing values by their standard deviation to overcome scaling and interpretability issues, and extracted their lower triangles (since all matrices were symmetrical). Then we regressed the dependent variable on our predictors, including nation identities as random effects. The full model including linguistic, religious and geographical connections between nations can be expressed as follows:2
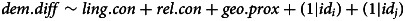
where *dem.diff* represents pairwise difference in democracy between pairs of nations, *ling.con* represents the nations’ linguistic connections, *rel.con* represents their religious connections, *geo.prox* is their geographical proximity and (1|*id_i_*) and (1|*id_j_*) are the random effects for the repeated identities of the nodes on each end of the dyadic relationship. We analysed all years available for each democracy measure (Freedom House, 1972–2020; Polity 5, 1800–2018; Vanhanen Index, 1810–2012), resulting in 466 cross-sectional analyses (Figure 3).

Statistical analysis was performed in R using the *lmer* function of the *lme4* package (Paradis et al., 2004). All statistical tests reported are two-sided.

### Contiguity

In order to control for simple contiguity (adjacency) effects, rather than effects of weighted cultural or geographical connections, we also constructed three contiguity matrices. Pairs of nations sharing a land or river border, the same majority language or the same majority religion were assigned a value of 1, and 0 in all other cases, leading to binary measures. For robustness checks, we repeated cross-sectional analyses adding to our models one contiguity matrix at a time (Figures S4–6).

### Longitudinal analysis

For each of the three democracy indicators, we predicted nations’ democracy scores at T2 from the weighted cumulative democracy score of their cultural relatives or geographical neighbours at T1, controlling for each nation's democracy at T1.

For this analysis we calculated three new predictor variables representing the weighted cumulative democracy of each nation's cultural and geographical neighbours at T1, as indicated by the linguistic, religious and geographical connection matrices. We multiplied each connection between a focal nation (a row in the matrix) and its neighbours (columns) with the democracy scores of the focal nation's neighbours at T1, and summed rows. These row sums gave an estimate of the cumulative democracy of a focal country's neighbours, weighted by their linguistic, religious or geographical proximity. The model can be expressed as follows:3
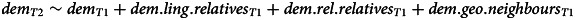
where *dem_T2_* and *dem_T1_* are the democracy scores at T2 and T1 respectively; *dem.ling.relatives_T1_* is the cumulative democracy of linguistic relatives at T1, weighted by respective linguistic connections; *dem.rel.relatives_T1_* is the cumulative democracy of religious relatives at T1, weighted by respective religious connections; *dem.geo.neighbours_T1_* is the cumulative democracy of geographical neighbours at T1, weighted by respective geographical proximities.

The appropriate time lag (separating T1 and T2) depends on the level of year-on-year variation in the democracy values and probable speed with which values are likely to spread between nations. We report findings based on a 10-year lag but also ran shorter (5 year) and longer (20 year) lags as robustness checks (see Figures S7–8). Lags smaller than 5 years were too highly correlated with the outcome variable (democracy at T2) and as a result caused model convergence issues.

### Explained variance

Although estimating coefficients of determination (*R*^2^) in mixed models is challenging, they can give a good general indication of the magnitude of an observed relation independent of sample size and complement significance testing (Selya et al., 2012). To estimate the explained variance, we calculated semi-partial coefficients of determination for each fixed effect after removing the variance explained by other predictors using the *r2beta* function of the *r2glmm* package (Jaeger, 2017). We relied on the Nakagawa approach (Nakagawa et al., 2017), which is appropriate for data of this structure.

### Alternative cultural connection measures

To evaluate the robustness of findings to choice of proximity metrics, we produced alternative versions of our cultural networks. We kept everything in our methodology constant, except for the way branch lengths in our language and religion trees, and consequently distances between languages and religions, were calculated. In our alternative approach, we set the branch lengths of the trees equal to 1 and then calculated relatedness between taxa using a patristic distance approach (Eff, 2008). Hence, similarity *s* between languages *i* and *j* was set as the distance *d* (in number of edges) from their most recent common ancestor *m* to the root of the tree *r*, standardised by the height of the tree *d_r_*, through the formula:4
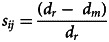
where *d_r_* is the maximum path length (in number of edges) from any taxon to the root (tree height) and *d_m_* is the maximum path length from any taxon to the most recent common ancestor *m*. Calculations were made in R using the packages *ape* (Paradis et al., 2004), *phytools* (Revell, 2012) and *phangorn* (Schliep, 2011). This approach resulted in new measures *s_ij_* for equation (1), producing two alternative matrices of linguistic and religious connections between nations.

Model selection using the corrected Akaike Information Criterion indicated that the connection measures used in our main analyses were always preferred over the alternative measures over the last ~50 years for which we have data on all three democracy measures and the largest sample of countries (1972–2020; see Figure S9). For periods prior to that, either the alternative networks were preferred or both models performed equally well. See the Supplementary Materials for results based on these networks.

### Regression assumptions

**Figure 4.**
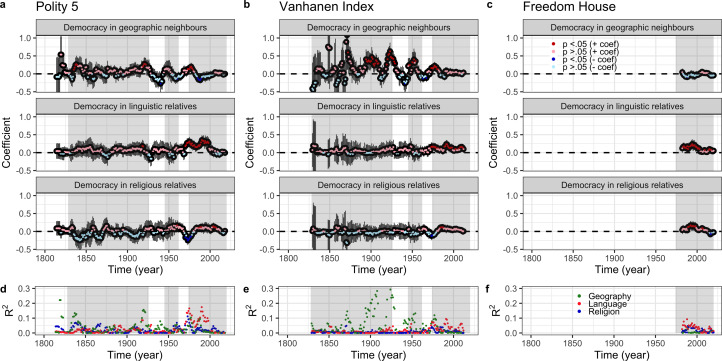
Independent effects of democracy among geographical, linguistic, and religious connections at T1 predicting democracy at T2 (10 year lag). Nations’ democracy scores at T2 were simultaneously regressed on the cumulative democracy of their geographical, linguistic and religious connections at T1 (10 years prior), after controlling for their democracy at T1. These analyses essentially trace changes in democracy over a 10-year period based on democracy in neighbouring or related nations (see also Figure 2). Each time slice was analysed separately for each of the three democracy measures (see Methods), resulting in 426 longitudinal models. Multiple regression standardised coefficients of the three main predictors are presented separately for Polity 5 (a, 1810–2018), the Vanhanen Index (b, 1820–2012) and Freedom House data (c, 1982 –2020), with 95% CI annotated. The direction and significance of effects are colour-coded: red for significant positive coefficients (*p* < 0.05), pink for non-significant positive coefficients, dark blue for significant negative coefficients (*p* < 0.05) and light blue for non-significant negative coefficients. (d–f) Semi-partial coefficients of determination (*R*^2^) are displayed below the respective models and outcome variables from a-c, indicating the proportion of variance in democracy explained by the cumulative democracy of geographical neighbours (green), and linguistic (red) or religious relatives (blue), after controlling for the other two variables and difference in democracy at T1. The three waves of democratisation are highlighted in grey on all graphs.

In order to evaluate departures from the regression assumptions of homogeneity of variance and normality of residuals, we examined residual plots and quantile–quantile plots (Q–Q plots), sampling every five years, out of a pool of 893 core cross-sectional and longitudinal analyses (corresponding to the results in Figure 3 and 4) – see Figures S10–21. Regarding the cross-sectional analyses, the oblique, band-like distribution of fitted values against residuals is typical for discrete dependent variables (such as our differences in democracy indices), which have a hard upper and lower limit for values (Turchin, 2018). What matters is that values are centred around zero, which is largely confirmed across the bulk of the range of fitted values by locally weighted smoothing. The Q–Q plots indicate that the assumption of normality is met more clearly in some years compared with others, suggesting that some caution is necessary when interpreting these parameter estimates. Residual and Q–Q plots generally look better for the longitudinal analyses, although again, suggest some caution is necessary when interpreting these estimates. One approach to deal with these departures from normality would be to transform the data or fit regression models using different likelihood functions (e.g. beta or ordinal regression). However, because the observed departures from normality are variable in size and direction, this would require different transformations and/or likelihood functions across variables and years, undermining the comparability of parameter estimates across democracy indicators and time. Moreover, our results remain consistent across predictor variables, various transformations of the data and robustness checks, mitigating concerns about the sensitivity of our findings to specific violations of modelling assumptions. We have therefore opted to retain the Gaussian likelihood function, but present our residual and Q–Q plots for transparency.

## Results

### National democratic indicators are more similar among cultural relatives

For each time slice in our dataset, we first ran a series of bivariate dyadic regressions (Karimov & Matthews, 2017) predicting between-country differences in each democracy indicator separately from each of our measures of linguistic, religious, and geographical connection (see Methods). These analyses revealed that linguistic, religious and geographical connections can all separately predict the three democracy indicators for at least part of the 220-year period covered by our data (Figure S22a–c). Standardised coefficients (which are comparable across time, predictors and democracy measures) indicate that, while effects varied through time, when present, linguistic and religious effects were generally as strong as or stronger than geographical effects. Over the last 50 years, which corresponds roughly to the third wave of democratisation and during which we have data on all three democracy measures across a large sample of countries, linguistic and religious connections are particularly important and consistent predictors, accounting for up to 17.6% and 25.5% of the variance in pairwise differences in democracy, respectively, compared with 5.4% for geographical proximity (Figure S22d–f).

Since linguistic, religious and geographical connections are themselves correlated (Table S2), we next sought to quantify and control for the independent effects of each predictor by including all three connection measures in dyadic regressions predicting pairwise differences in democracy indicators at each time slice. These analyses revealed independent effects of all three predictors on each democracy indicator over the time period covered by our data (Figure 3a–c). While the effects of linguistic and religious ancestry were slightly attenuated in this combined model, when present, they remained generally better predictors of all three democracy indicators than geography. Over the last 50 years, linguistic and religious ancestry accounts for up to 12.3% and 17.4% of variance in pairwise differences in democracy, respectively, compared with 1.2% for geographical proximity (see also Figure 3d–f).

Figure 3 also highlights clear variation in the importance of each of the three predictors both within and between Huntington's waves of democratisation. Intriguingly, controlling for geography, we find little consistency through time in the effects of linguistic or religious ancestry over the course of the first wave. However, across all three democracy indicators, linguistic ancestry is an increasingly important predictor beginning mid-way through the second wave (circa 1955) and plateauing (or, in the case of Polity 5, declining somewhat) in the third wave from about 1990 to the present. Likewise, religious ancestry becomes an increasingly important predictor of similarity in all three democracy measures from approximately the beginning of the third wave, circa 1975, plateauing and then declining somewhat from circa 2000 to the present. Shared religious ancestry between nations also predicts that their Polity 5 scores will be more similar during a brief period from 1917 to 1928. It is also worth noting a brief negative effect of religious ancestry during the mid–nineteenth century for Polity 5 and early the twentieth century for the Vanhanen Index. Given that no such effect is present in the bivariate religion analyses (Figure S22a–b), we suspect that this is an artefact of multicollinearity resulting from the small sample size during these time windows (30–55 nations) together with correlations between geographical, linguistic and religious connections for the sampled nations.

Figure 3 shows a consistently positive correlation between geographical proximity and the similarity of democratic outcomes, although this varies in magnitude through time and across the different democracy indicators. Geographic proximity becomes an increasingly important predictor of the Vanhanen Index over the course of the first wave, until approximately 1925, when the importance of geography starts to attenuate. By the time of the third wave, geographical proximity explains a small but statistically significant proportion of variance in the Vanhanen Index, a pattern repeated in the third wave data available from Freedom House. The effects of geographical proximity on Polity 5 scores show a different pattern, with a strong effect declining in the first half of the nineteenth century and, as we see for religious ancestry, a brief resurgence at the end of the first wave.

These findings are consistent with the proposal that global variation in democracy reflects the influence of both spatial diffusion and cultural barriers to diffusion tied to deep cultural ancestry. However, one alternative explanation for the patterns we observe is that they are an artefact of common language or religion shared by modern populations (e.g. the Anglophone world or the Islamic world) and do not reflect the deeper, cross-cutting cultural relationships captured by our ancestral cultural connection measures. Similarly, the effect of geographical proximity that we observe could be due to the influence of shared borders, rather than geographical proximity *per se*. To quantify and control for these effects, we repeated the above analyses including an additional linguistic, religious or geographical contiguity matrix as a predictor, i.e. a binary matrix indicating whether nation pairs share a majority language, majority religion or border. While linguistic contiguity, and to a lesser extent geographical contiguity, did predict democracy indicators for some time periods, including these contiguity measures in our model had no appreciable effect on our estimates of the importance of linguistic or religious ancestry, or geographical proximity through time (Figures S4–6). Interestingly, linguistic contiguity was a significant predictor of Polity 5 and Vanhanen Index scores throughout the first and second waves of democratisation, complementing and then apparently being replaced by the effect of ancestral linguistic relationships (Figure S5).

We also wanted to explore how robust our findings were across different methods of calculating cultural and geographical connections. We therefore ran the analyses summarised in Figure 3 using alternative measures of linguistic, religious and geographical connections (see Methods). We found that using unlogged geographical proximity reduced the importance of geography as a predictor of democracy indicators but had no appreciable effect on the importance of our cultural measures (Figure S23). Using alternative metrics of cultural ancestry had no impact on the recent importance of religious ancestry, but did impact the effect of linguistic ancestry (Figure S24), such that linguistic ancestry showed a stronger effect in the mid-nineteenth and early to mid-twentieth centuries for the Polity 5 and Vanhanen Indices, becoming relatively less important during the third wave.

### Diffusion of democracy between cultural relatives

The above analyses consider the pattern of variation in democratic outcomes among cultural relatives and geographical neighbours at a given point in time. Next we consider the cultural contagion of democracy through time – i.e. whether the democratic status of a target nation at a particular point in time is predicted by its own prior democratic status as well as the democratic status of its cultural relatives or geographical neighbours. To do this, we regressed nations’ democracy scores at Time 2 (T2) on the weighted cumulative democracy scores of their cultural relatives or geographical neighbours at T1 (10 years previous; see Methods for more details), controlling for nations’ democracy scores at T1.

Figure 4 shows how the effects of the prior democratic status of nations’ cultural relatives or geographical neighbours vary through time and across democracy indicators. The democratic status of nations’ linguistic relatives is the only effect to show a consistently positive trend across all three democratic outcome measures for the duration of the time series. The language ancestry effect is strongest, and statistically significant for most of the third wave of democratisation across all outcome measures. Unsurprisingly, since democratic status tends to persist, most of the variation in nations’ democracy indicators at T2 is explained by their democracy at T1, but linguistic ancestry accounts for a non-trivial component of the remaining variation, explaining up to 17.4% of variation across outcome measures in the third wave. The democratic status of nations’ religious relatives shows no consistent effect until the third wave, when we see a sustained positive trend across all outcome measures, consistent with our cross-sectional analyses, with religious ancestry explaining up to 11.1% of the variation in democratic outcomes during this period. Also in accordance with our cross-sectional analyses, the effects of a nation's geographical neighbours on its democratic outcomes tend to be positive, although these geographical effects show more variation through time and across outcome measures. Most notably, we see evidence of punctuated periods of geographical diffusion for the Vanhanen Index, with strong positive effects of geographical neighbours at the turn of the twentieth century and for intervals spanning the First and Second World Wars, with geography explaining up to 37% of the variation. In contrast, there is no evidence for an effect of a nation's geographical neighbours on later democratic outcomes for the Freedom House measure.

As for our cross-sectional findings, these longitudinal results are robust to variations in modelling assumptions. Repeating the analyses using our alternative cultural and geographical metrics did not substantially alter the relationships we observe, although geographical proximity was slightly less important (and showed less variability in coefficients of determination; Figures S25–26). In line with the cross-sectional analyses, we find that using our alternative measures of cultural relatedness, the importance of linguistic ancestry as a predictor of change in the Vanhanen Index expands to cover most of the twentieth century (Figure S26). Finally, to test whether our findings were robust across different choices of time lag, we repeated the above analyses with shorter (5 year) or longer (20 year) time lag (see Methods). These additional analyses revealed similar patterns to Figure 4 (Figures S7–8), although we see more statistically significant effects in the 20-year lag analysis and fewer using a 5-year lag. This suggests that relatively slow rates of democratic change generate less signal for shorter intervals, but the impact of democratic outcomes in a nation's relatives is enduring enough that they can predict its democratic trajectory even decades later.

## Discussion

Our findings show that political changes among nations are not independent events, playing out along isolated trajectories. Rather, they form clusters that reflect both cultural and geographical connections between nations. We find that the effects of linguistic, religious and geographical connections vary substantially through time, but nevertheless wax and wane in a manner that is non-random. Where linguistic, religious, and geographical effects are present, they are almost always positive – i.e. democracy indicators consistently tend to be more similar, rather than more divergent, among linguistic or religious relatives and geographical neighbours. Our multiple regression analyses indicate that the effects of linguistic and religious ancestry are independent of geographical connections between nations and, particularly in recent times, are at least as important as geographical effects – language ancestry explains up to about 12.3% and religious ancestry as much as 17.4% of the variance in democratic indicators. In addition, these broad patterns hold across three different democracy indicators spanning many decades and are robust to variation in the specific network metrics employed. Further, the linguistic and religious ancestry effects we observe are not weakened by the inclusion of common language or religion in our models, suggesting they reflect the impact of deeper cultural ties between nations, rather than simply a particular shared language or religion today.

We also find evidence for the diffusion of democratic outcomes between nations over time. We show that, particularly over the last half century, it is possible to predict a nation's future democratic trajectory based on the democratic status of its linguistic and, less clearly, religious relatives, that these cultural effects are at least as important as geographical neighbourhood, and that they may persist for decades. This supports a ‘cultural contagion’ model and ‘cultural barrier effects’ (Spolaore & Wacziarg, 2009), in which democratic outcomes preferentially diffuse between cultural relatives, either because they are more likely to share social and economic connections or because they are more similar and hence likely to respond to innovations and events in similar ways.

Examining variation in the independent effects of linguistic, religious and geographical connections through time reveals several intriguing trends. Over the course of Huntington's first wave of democratisation, up to and including the years following the First World War, our sample of nations is small (~68 or fewer), and so our findings need to be interpreted with caution. Nevertheless, there is evidence for geographical clustering of democratic status prior to 1850 in the case of Polity 5 and in the decades after 1850 in the Vanhanen Index, possibly reflecting the erosion of executive power in European monarchies and gradual emergence of systems allowing electoral participation and competition in the wake of the 1848 ‘Spring of Nations’. We also see an increase in the importance of geography as a predictor of the Vanhanen Index between 1918 and 1926, and a concomitant spike in the importance of geography and religion as predictors of Polity 5 during the same window, probably tied to the geo-political divisions that emerged in the aftermath of the First World War.

From the second half of the twentieth century and into the twenty-first century, our analysis indicates that deep cultural ancestry has become a more important predictor of all three democratic outcome measures. These findings support Huntington's (1991) proposal that in the third wave of democratisation (beginning in 1974) ethnolinguistic and religious differences became increasingly important forces shaping geo-political changes around the globe. Our analysis also suggests several additional insights. First, the rise in the predictive power of religious ancestry appears particularly abrupt (beginning only in circa 1975) and robust (holding across our alternative cultural connection measures), and most evident in the Freedom House indicator, which focuses primarily on individual rights and freedoms.

Second, it is interesting to note that shared linguistic ancestry appears to become more important somewhat earlier than the third wave, during the short second wave of democratisation (1945–1962), when international structural factors have typically been emphasised – namely the new international order that followed the Second World War and the process of decolonisation that saw former colonies adopt (sometimes fleetingly) the democratic institutions of their former colonisers.

Third, and relatedly, while Huntington himself emphasised the role of structural shifts in the third wave, such as economic development and US and EU foreign policy linked to the end of the Cold War, critics have argued that these factors were more important for some elements of democracy than others (e.g. Diamond, 2002; Schedler, 2002). Our findings indicate that, notwithstanding these structural factors, cultural ancestry and barrier effects (Spolaore & Wacziarg, 2009) play a role whether one considers restraints on executive function (Polity 5), electoral participation and competition (Vanhanen's Index) or freedom and individual rights (Freedom House). It is also worth noting that the cultural ancestry effects we see in the third wave are not simply an artefact of the post-communist transition to democracy among some eastern European countries in the 1990s. The increasing importance of linguistic ancestry begins well before the fall of communism, and the nations that emerged in the 1990s immediately following the fall of communism were not unusually democratic. While the trajectory of the Cold War was a crucial factor in global geopolitical change throughout the second half of the twentieth century, the fall of communism in Eastern Europe was only one component of the third wave, which also involved a succession of transitions in Western Europe, Latin America, the Pacific and sub-Saharan Africa.

Fourth, the trend Huntington identified in 1991 may be reversing. Across all three democratic indicators, the effect of religious ancestry appears to have peaked in the mid 2000s, although it remains at least as important a predictor as linguistic ancestry for the Freedom House and Polity 5 indices. The effect of linguistic ancestry also appears to be attenuating in the Polity 5 measures and when analysing the alternative proximity measures. It is worth noting that the increasing importance of cultural ancestry in our models post 1950 could be influenced by the larger sample of nations in more recent years (between 72 and 193 nations). We think this is unlikely, however, because no such pattern is observed for geographical proximity and, as we highlight above, the trend in the most recent years, when our sample is largest, has been for a slight decline in the importance of cultural ancestry.

A number of other limitations and caveats on our findings are worth noting. First, as with any empirical work, the validity of our conclusions is contingent on the general accuracy of our data – here, our measures of democratic status and cultural ancestry. While some scholars may disagree with particular democracy assignments or cultural relationships in our data, we think it highly unlikely that such errors would systematically bias our findings in favour of the hypotheses for which we find support, particularly across the three sets of democratic outcome measures and different estimates of linguistic and religious ancestry that we consider. More caution may be required when interpreting our failure to find cultural ancestry effects at earlier time periods for which our sample is smaller and less comprehensive. However, the similarity of the trends we observe across democracy measures makes arguments that missing data has greatly biased our findings more difficult to sustain. Second, it remains possible that some unmodelled ‘third variable’ accounts for the observed correlations that we observe between democratic outcomes and cultural ancestry. Controlling for geographical proximity as we do goes some way towards addressing possible ecological factors, which are themselves likely to be geographically auto-correlated. One might argue that unmodelled cultural traits may better account for the patterns we observe; however this is not inconsistent with the importance of cultural ancestry – as we note, democracy may diffuse between cultural relatives precisely because they share certain cultural traits owing to common ancestry. Third, we want to point out that while our findings can help us understand how nations achieve positive democratic outcomes, they do not speak to whether they can achieve such outcomes, which we see as within reach of all nations.

By combining data and tools from political science, cultural evolution and network science, our approach helps to explain enduring political clusters around the globe and promises a better understanding of the interplay between political outcomes, cultural ancestry and global networks. We have shown the longstanding cultural connections between nations captured by cultural genealogies of language and religion can help explain global variation in democracy and democratic change. Our findings complement prior research predicting democratic outcomes from country-level characteristics such as GDP per capita or primary productivity (e.g. Boix, 2011; Currie et al., 2021; Inglehart & Welzel, 2009; Knutsen et al., 2019). The cultural ancestry effects we identify do not support claims of cultural determinism, whereby the ultimate democratic prospects of a nation are tightly constrained by its cultural heritage. Nevertheless, they do indicate that, in describing global variation in democracy and the process of democratic change, culture matters. We anticipate that these findings may be of use to policy-makers seeking evidence-based information on the potential effects of cultural ancestry on the democratic trajectory of nations, and the role of cultural neighbours in shaping democratic change.

## Research transparency and reproducibility

Data and code for this study are available at the Open Science Framework repository https://osf.io/mhp76/

## Supplementary material

For supplementary material accompanying this paper visit https://doi.org/10.1017/ehs.2022.40. click here to view supplementary material
