# Supplementary material for: Shared cultural ancestry predicts the global diffusion of democracy
Source: Evol Hum Sci. 2022 Sep 19;4:e42. doi: 10.1017/ehs.2022.40 (PMC10426017; doi:10.1017/ehs.2022.40)
Supplement: Supplementary file 1 [file S2513843X22000408sup001.docx]

**Supplementary Materials for**

**Shared cultural ancestry predicts the global diffusion of democracy**

**Thanos Kyritsis, Luke Matthews, David Welch, and Quentin D. Atkinson***

* Correspondence concerning this article should be addressed to Quentin D Atkinson, School of Psychology, University of Auckland, Floor 2, Building 302, 23 Symonds Street, Auckland, 1010, New Zealand. E-mail: [q.atkinson@auckland.ac.nz](mailto:q.atkinson@auckland.ac.nz)

**
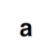
**

**
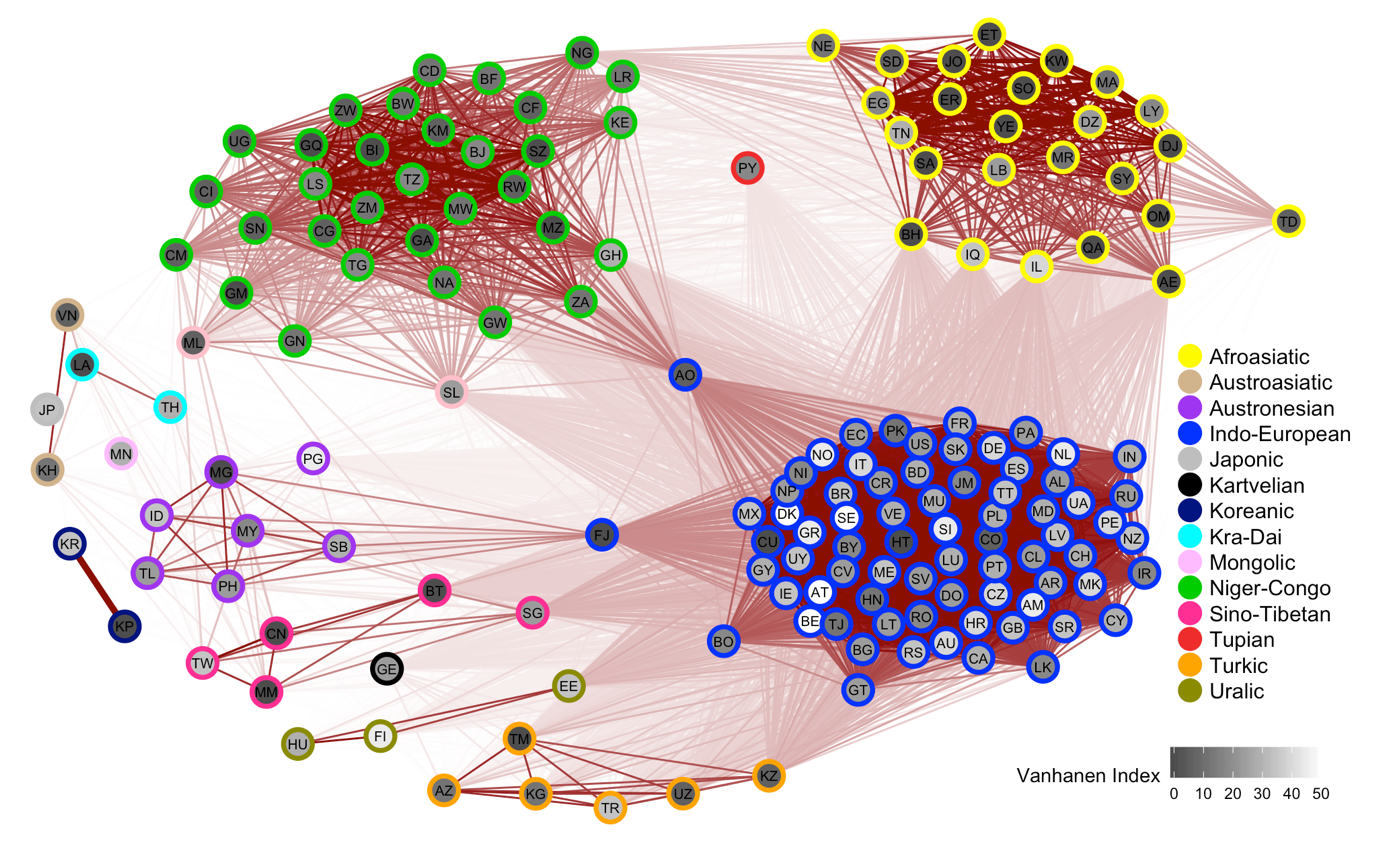
**

**
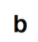
**

**
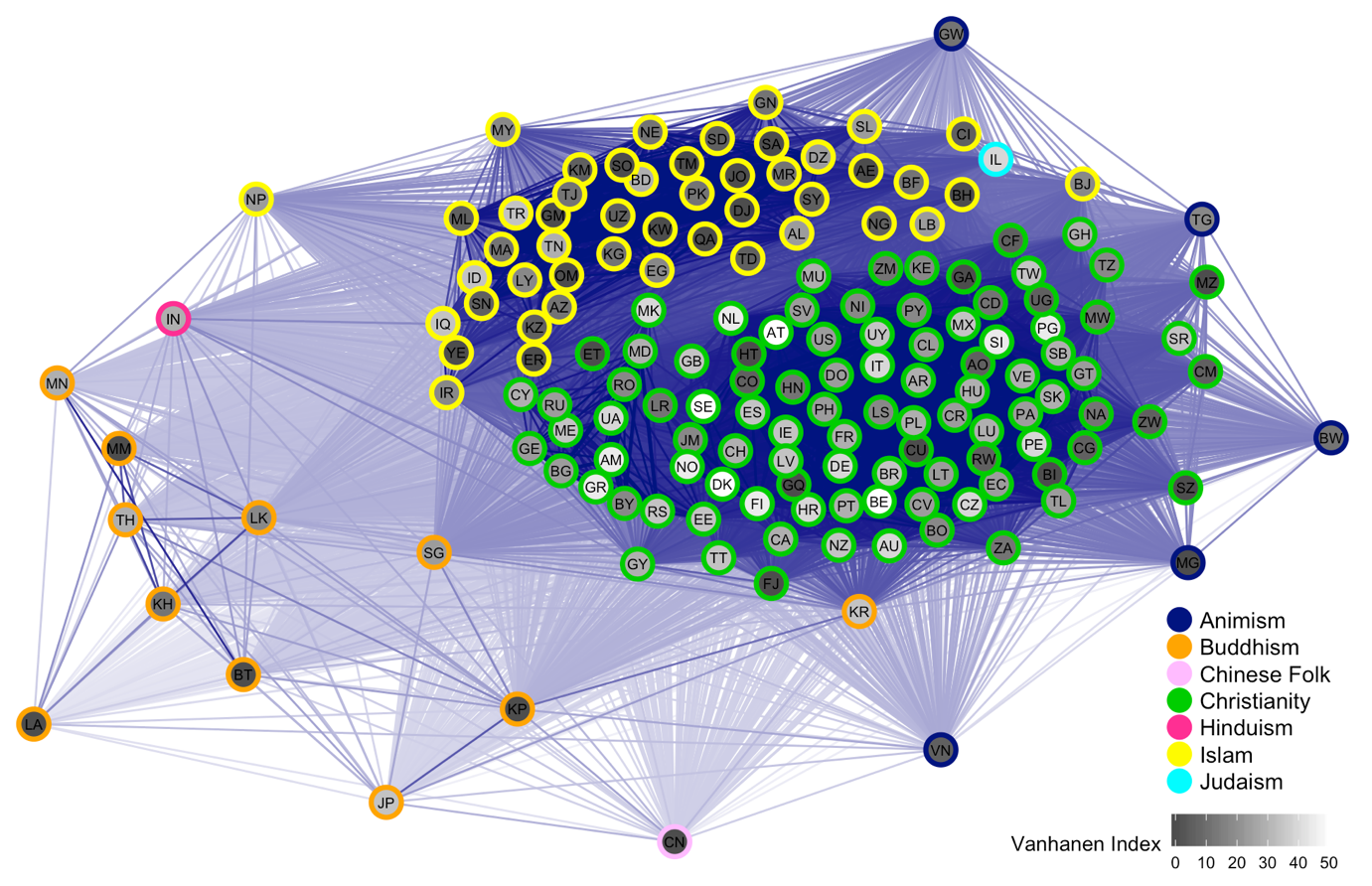
**

**Figure S1. Global variation in democracy (Vanhanen Index) across networks representing linguistic and religious connections between nations. (a)** Variation in Vanhanen Index scores for the year 2012 across a global network of linguistic connections (edges) between 163 contemporary nations (nodes). Lighter node hues indicate more democratic nations. Node proximity and edge transparency reflect linguistic connections based on all languages spoken by at least 1 permille of each nations’ population, weighted by their respective percentages (see Methods). Node borders are colour-coded by language family of the nation’s majority language (see Table 2 for assignments and ISO-codes). (**b**) As for (a) but showing religious connections based on percentage adherents to 28 major religions. Node borders are colour-coded by the nation’s majority religion (see Table S1 for assignments and ISO-codes).

**
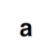

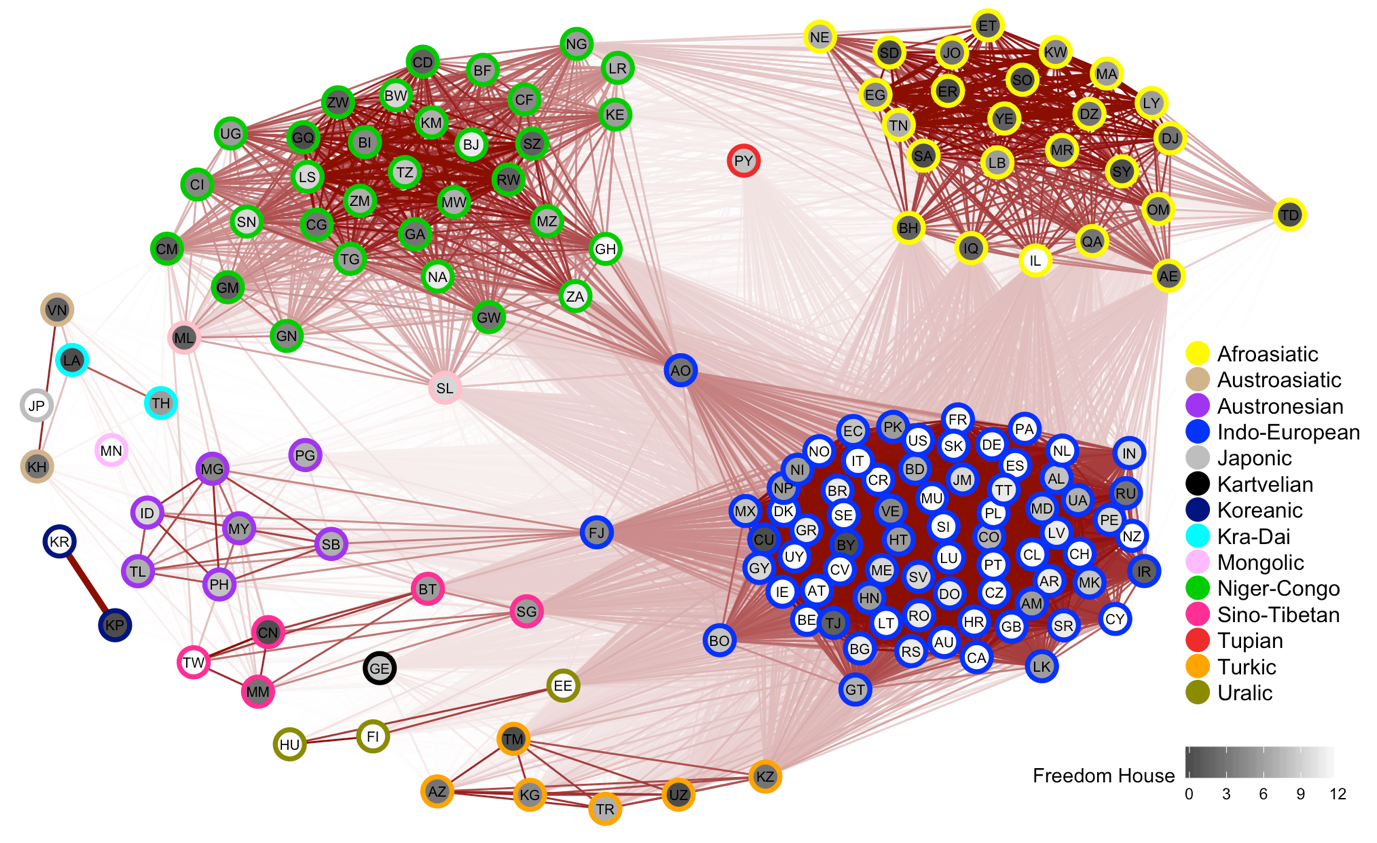
**

**
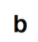
**

**
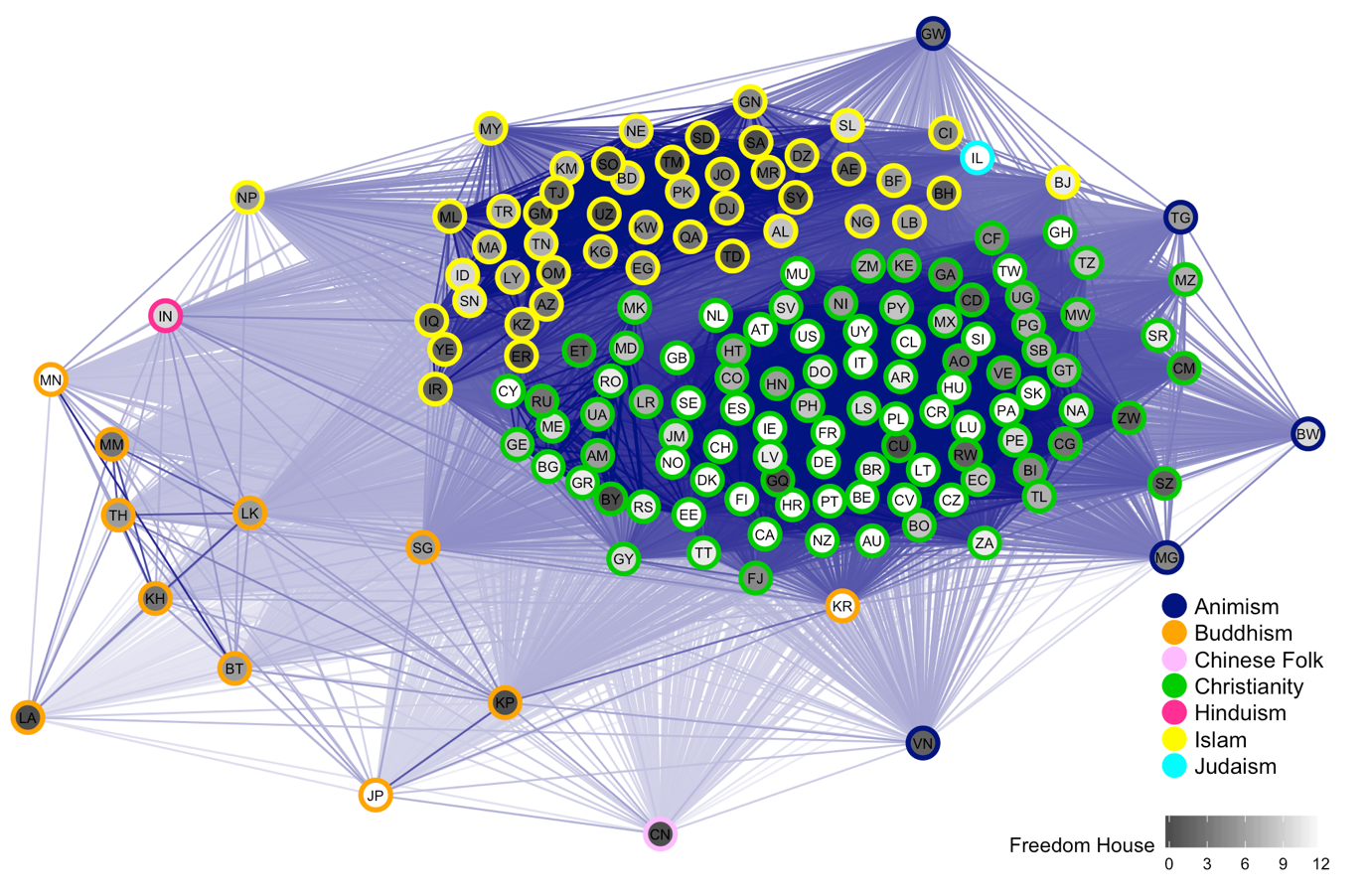
**

**Figure S2. Global variation in democracy (Freedom House) across networks representing linguistic and religious connections between nations. (a)** Variation in Freedom House scores for the year 2012 across a global network of linguistic connections (edges) between 163 contemporary nations (nodes). Lighter node hues indicate more democratic nations. Node proximity and edge transparency reflect linguistic connections based on all languages spoken by at least 1 permille of each nations’ population, weighted by their respective percentages (see Methods). Node borders are colour-coded by language family of the nation’s majority language (see Table 2 for assignments and ISO-codes). (**b**) As for (a) but showing religious connections based on percentage adherents to 28 major religions. Node borders are colour-coded by the nation’s majority religion (see Table S1 for assignments and ISO-codes).


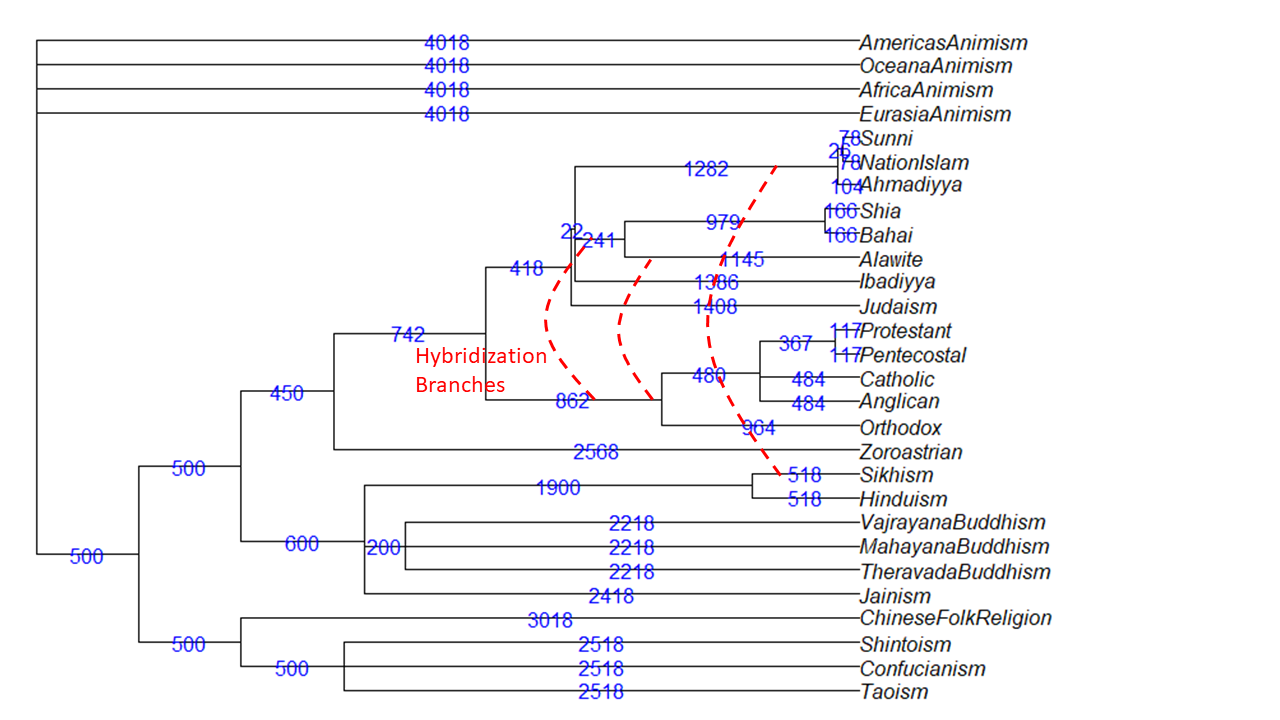


**Figure S3. Dated ultrametric religion tree.** This tree was used for the analyses presented in the main paper (Fig. 2-4). Numbers (in blue) represent branch lengths in years. Red dotted lines indicate hybridization events. Divergence times are based on data sources in Supplementary Methods below.

**Figure S4. Independent effects of geographic, linguistic, and religious connections predicting democracy (including geographic contiguity).** Pairwise differences in democracy between nations were simultaneously regressed on geographic, linguistic and religious connections between nations, as well as their geographic contiguity, at each time slice for which data was available, resulting in 466 cross-sectional models. Multiple regression standardized coefficients of the three predictors are presented separately for Polity 5 (**a**; 1800-2018), the Vanhanen Index (**b**; 1810-2012), and Freedom House data (**c**; 1972-2020), with 95% CI annotated. The direction and significance of effects colour-coded: red for significant positive coefficients (p < .05), pink for non-significant positive coefficients, dark blue for significant negative coefficients (p < .05), and light blue for non-significant negative coefficients. **d-f**, Semi-partial coefficients of determination (R^2^) are displayed below the respective models and outcome variables from a-c, indicating the proportion of variance in democracy explained by geographic contiguity (light green) geography (green), language (red), or religion (blue), after controlling for the other three variables. The three waves of democratization are highlighted in gray on all graphs.

**Figure S5. Independent effects of geographic, linguistic, and religious connections predicting democracy (including linguistic contiguity).** Pairwise differences in democracy between nations were simultaneously regressed on geographic, linguistic and religious connections between nations, as well as their linguistic contiguity, at each time slice for which data was available, resulting in 466 cross-sectional models. Multiple regression standardized coefficients of the three predictors are presented separately for Polity 5 (**a**; 1800-2018), the Vanhanen Index (**b**; 1810-2012), and Freedom House data (**c**; 1972-2020), with 95% CI annotated. The direction and significance of effects colour-coded: red for significant positive coefficients (p < .05), pink for non-significant positive coefficients, dark blue for significant negative coefficients (p < .05), and light blue for non-significant negative coefficients. **d-f**, Semi-partial coefficients of determination (R^2^) are displayed below the respective models and outcome variables from a-c, indicating the proportion of variance in democracy explained by linguistic contiguity (yellow), geography (green), language (red), or religion (blue), after controlling for the other three variables. The three waves of democratization are highlighted in gray on all graphs.

**Figure S6. Independent effects of geographic, linguistic, and religious connections predicting democracy (including religious contiguity).** Pairwise differences in democracy between nations were simultaneously regressed on geographic, linguistic and religious connections between nations, as well as their religious contiguity, at each time slice for which data was available, resulting in 466 cross-sectional models. Multiple regression standardized coefficients of the three predictors are presented separately for Polity 5 (**a**; 1800-2018), the Vanhanen Index (**b**; 1810-2012), and Freedom House data (**c**; 1972-2020), with 95% CI annotated. The direction and significance of effects colour-coded: red for significant positive coefficients (p < .05), pink for non-significant positive coefficients, dark blue for significant negative coefficients (p < .05), and light blue for non-significant negative coefficients. **d-f**, Semi-partial coefficients of determination (R^2^) are displayed below the respective models and outcome variables from a-c, indicating the proportion of variance in democracy explained by religious contiguity (cyan) geography (green), language (red), or religion (blue), after controlling for the other three variables. The three waves of democratization are highlighted in gray on all graphs.

**Figure S7. Independent effects of democracy among geographic, linguistic, and religious connections at T1 predicting democracy at T2 (5-year lag).** Nations’ democracy scores at T2 were simultaneously regressed on the cumulative democracy of their geographic, linguistic and religious connections at T1 (5 years prior), after controlling for their democracy at T1. These analyses essentially trace changes in democracy over a 5-year period based on democracy in neighbouring or related nations (see also Fig. 2). Each time-slice was analysed separately for each of the three democracy measures (see Methods), resulting in 456 longitudinal models. Multiple regression standardized coefficients of the three main predictors are presented separately for Polity 5 (**a**; 1805-2018), the Vanhanen Index (**b**; 1815-2012), and Freedom House data (**c**; 1977-2020), with 95% CI annotated. The direction and significance of effects colour-coded: red for significant positive coefficients (p < .05), pink for non-significant positive coefficients, dark blue for significant negative coefficients (p < .05), and light blue for non-significant negative coefficients. **d-f**, Semi-partial coefficients of determination (R^2^) are displayed below the respective models and outcome variables from a-c, indicating the proportion of variance in democracy explained by the cumulative democracy of geographic neighbours (green), and linguistic (red) or religious relatives (blue), after controlling for the other two variables and difference in democracy at T1. The three waves of democratization are highlighted in gray on all graphs.

**Figure S8. Independent effects of democracy among geographic, linguistic, and religious connections at T1 predicting democracy at T2 (20-year lag).** Nations’ democracy scores at T2 were simultaneously regressed on the cumulative democracy of their geographic, linguistic and religious connections at T1 (20 years prior), after controlling for their democracy at T1. These analyses essentially trace changes in democracy over a 20-year period based on democracy in neighbouring or related nations (see also Fig. 2). Each time-slice was analysed separately for each of the three democracy measures (see Methods), resulting in 410 longitudinal models. Multiple regression standardized coefficients of the three main predictors are presented separately for Polity 5 (**a**; 1820-2018), the Vanhanen Index (**b**; 1830-2012), and Freedom House data (**c**; 1992-2020), with 95% CI annotated. The direction and significance of effects colour-coded: red for significant positive coefficients (p < .05), pink for non-significant positive coefficients, dark blue for significant negative coefficients (p < .05), and light blue for non-significant negative coefficients. **d-f**, Semi-partial coefficients of determination (R^2^) are displayed below the respective models and outcome variables from a-c, indicating the proportion of variance in democracy explained by the cumulative democracy of geographic neighbours (green), and linguistic (red) or religious relatives (blue), after controlling for the other two variables and difference in democracy at T1. The three waves of democratization are highlighted in gray on all graphs.


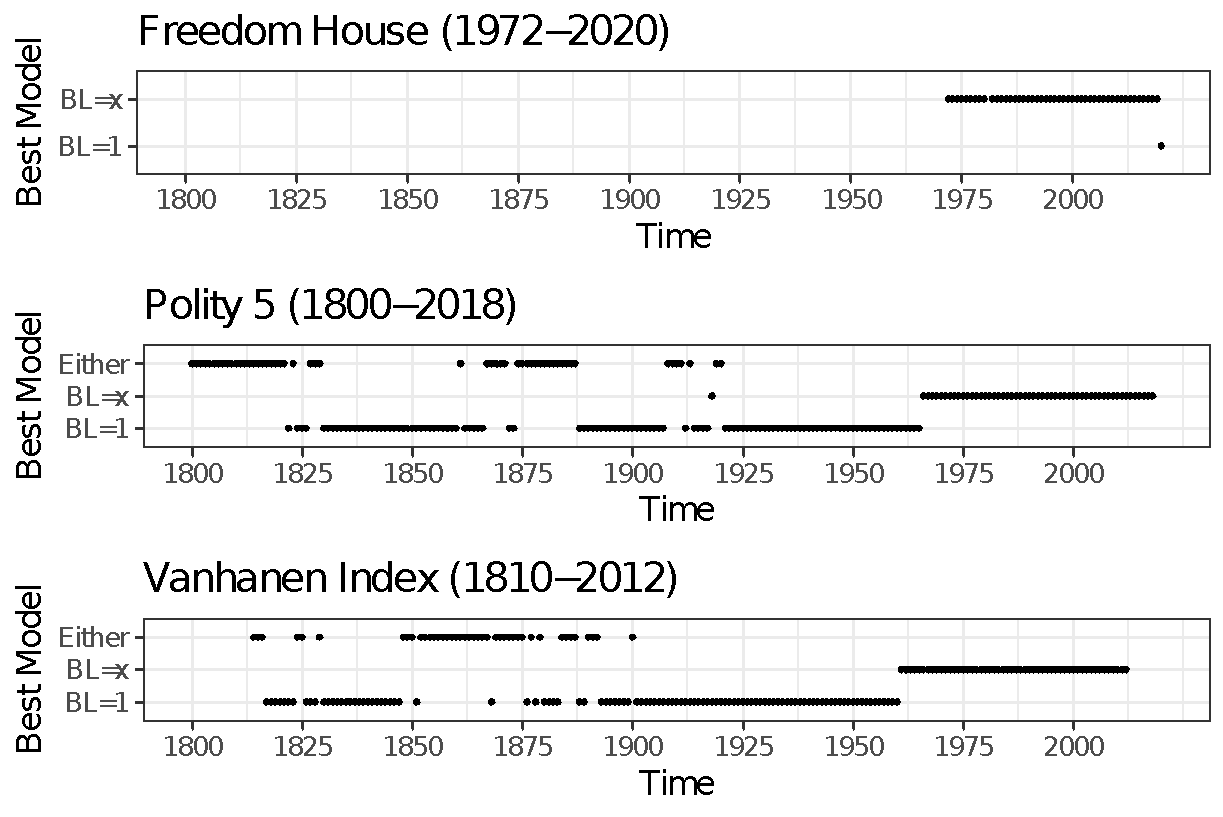


**Figure S9. Cultural predictor selection for democracy indicators.** Preferred cultural predictors of each democracy indicator by year, based on corrected Akaike Information Criterion (AICc) selection. We compared two sets of cross-sectional models, controlling for geographic proximity, that included linguistic and religious connections based on either branch lengths that produce ultrametric cultural phylogenies (BL=x) or cladograms with uniform branch lengths (BL=1) (see Methods). Selection was based on a difference of AICc < 4 units from the preferred model, which is a threshold frequently used to indicate that these predictors have almost the same performance (Burnham & Anderson, 2004). Over the last ~50 years for which we have data on all three democracy measures and the largest sample of countries (1972-2020) the connections based on ultrametric cultural phylogenies were preferred. For periods prior to that, either the connections based on uniform branch lengths were preferred or both models performed equally well.


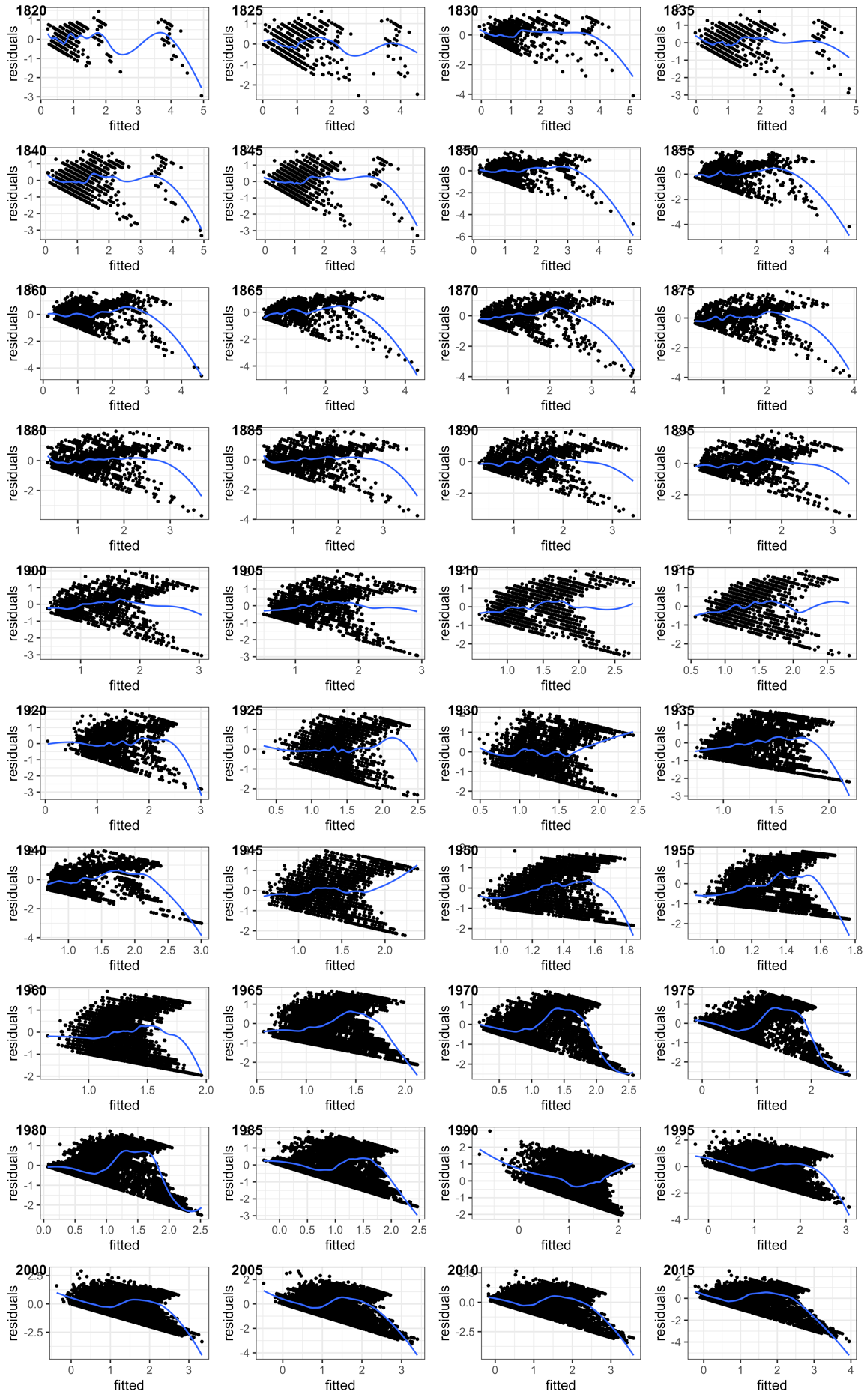


**Figure S10. Residuals against fitted values for cross-sectional analysis of Polity 5 data.** For description of the models and interpretation of residual plots see Methods; for results of these models see Fig. 3.


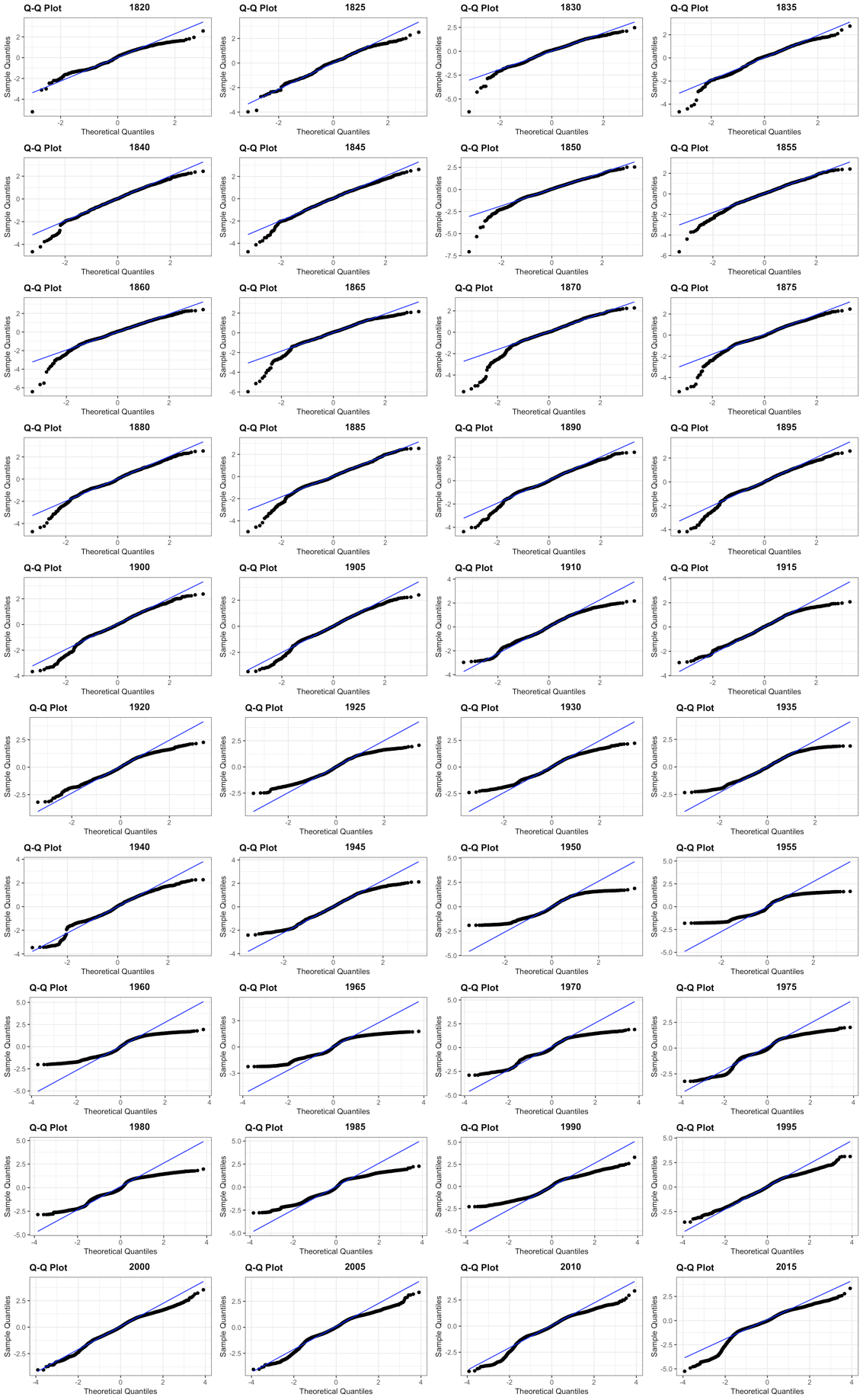


**Figure S11. Q-Q plots for the cross-sectional analysis of Polity 5 data.** For description of the models and interpretation of Q-Q plots see Methods; for results of these models see Fig. 3.


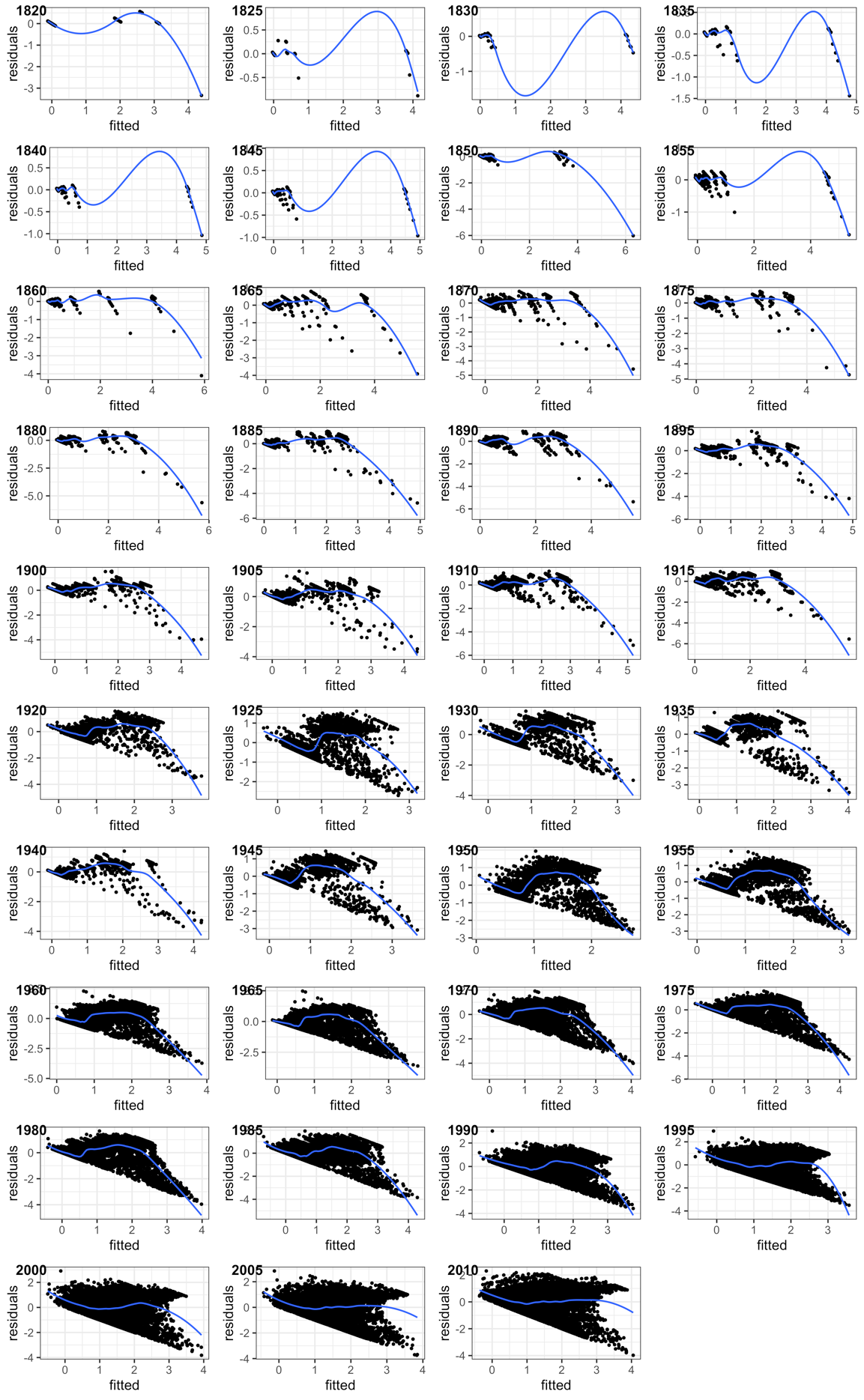


**Figure S12. Residuals against fitted values for cross-sectional analysis of Vanhanen data.** For description of the models and interpretation of residual plots see Methods; for results of these models see Fig. 3.


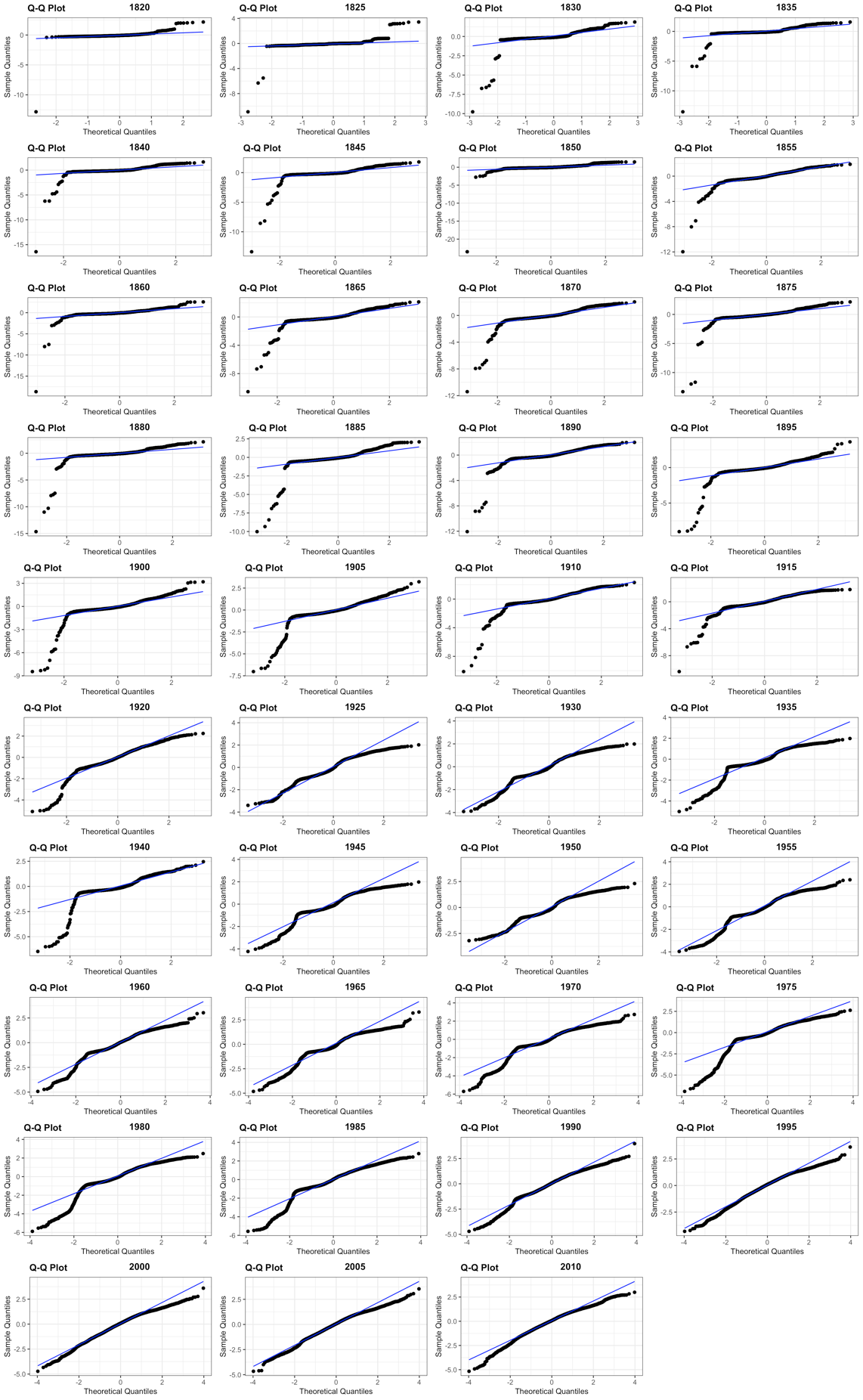


**Figure S13. Q-Q plots for the cross-sectional analysis of the Vanhanen Index.** For description of the models and interpretation of Q-Q plots see Methods; for results of these models see Fig. 3.


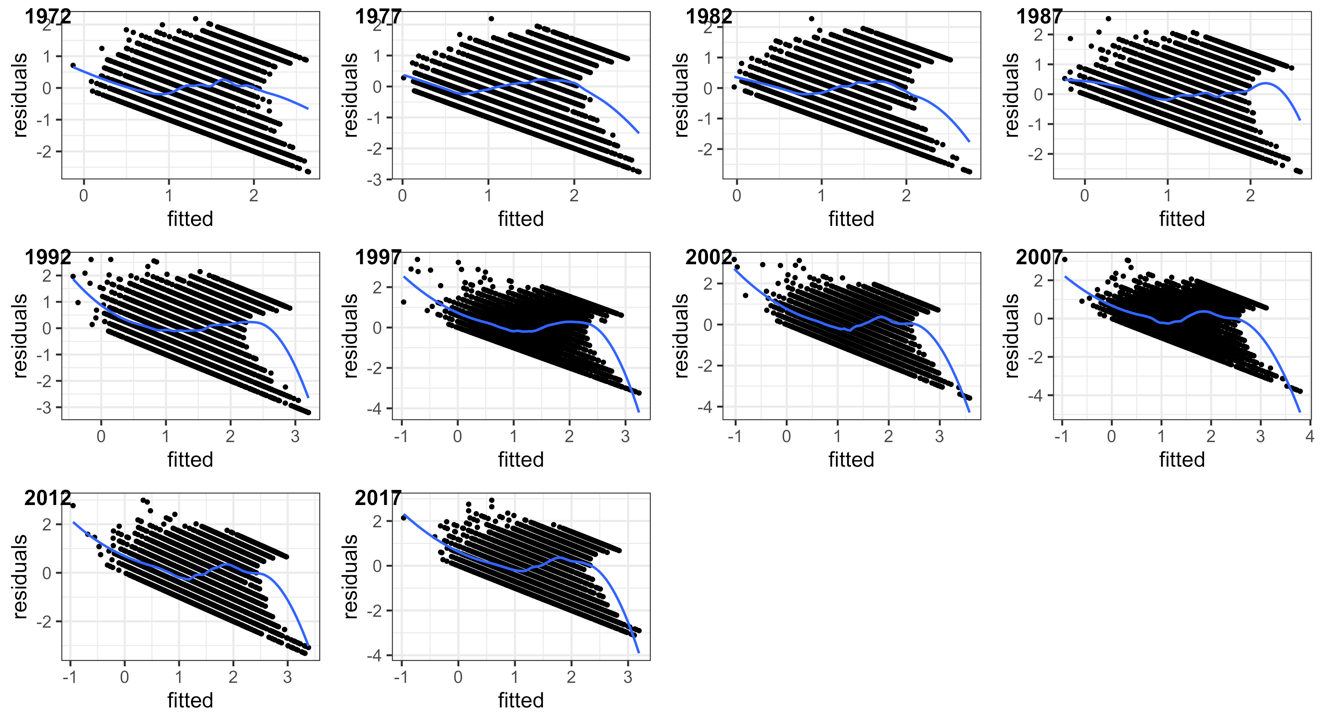


**Figure S14. Residuals against fitted values for cross-sectional analysis of Freedom House data.** For description of the models and interpretation of residual plots see Methods; for results of these models see Fig. 3.


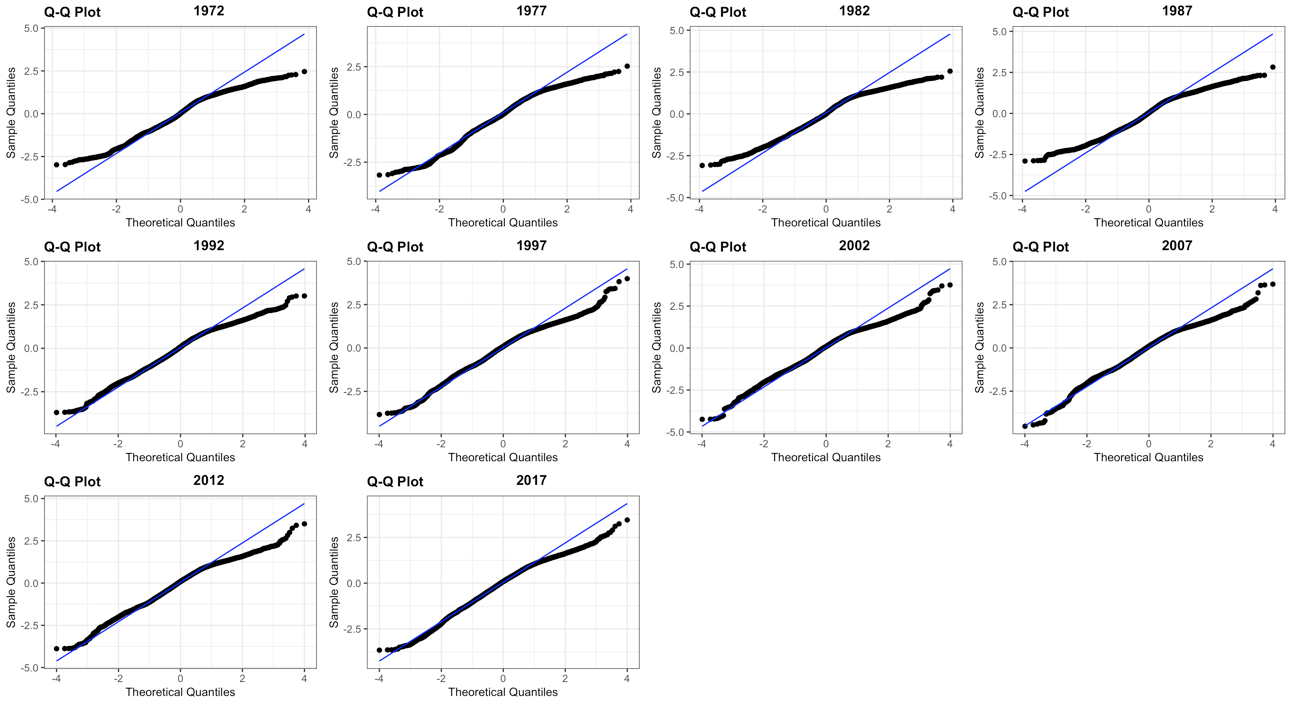


**Figure S15. Q-Q plots for the cross-sectional analysis of Freedom House data.** For description of the models and interpretation of Q-Q plots see Methods; for results of these models see Fig. 3.


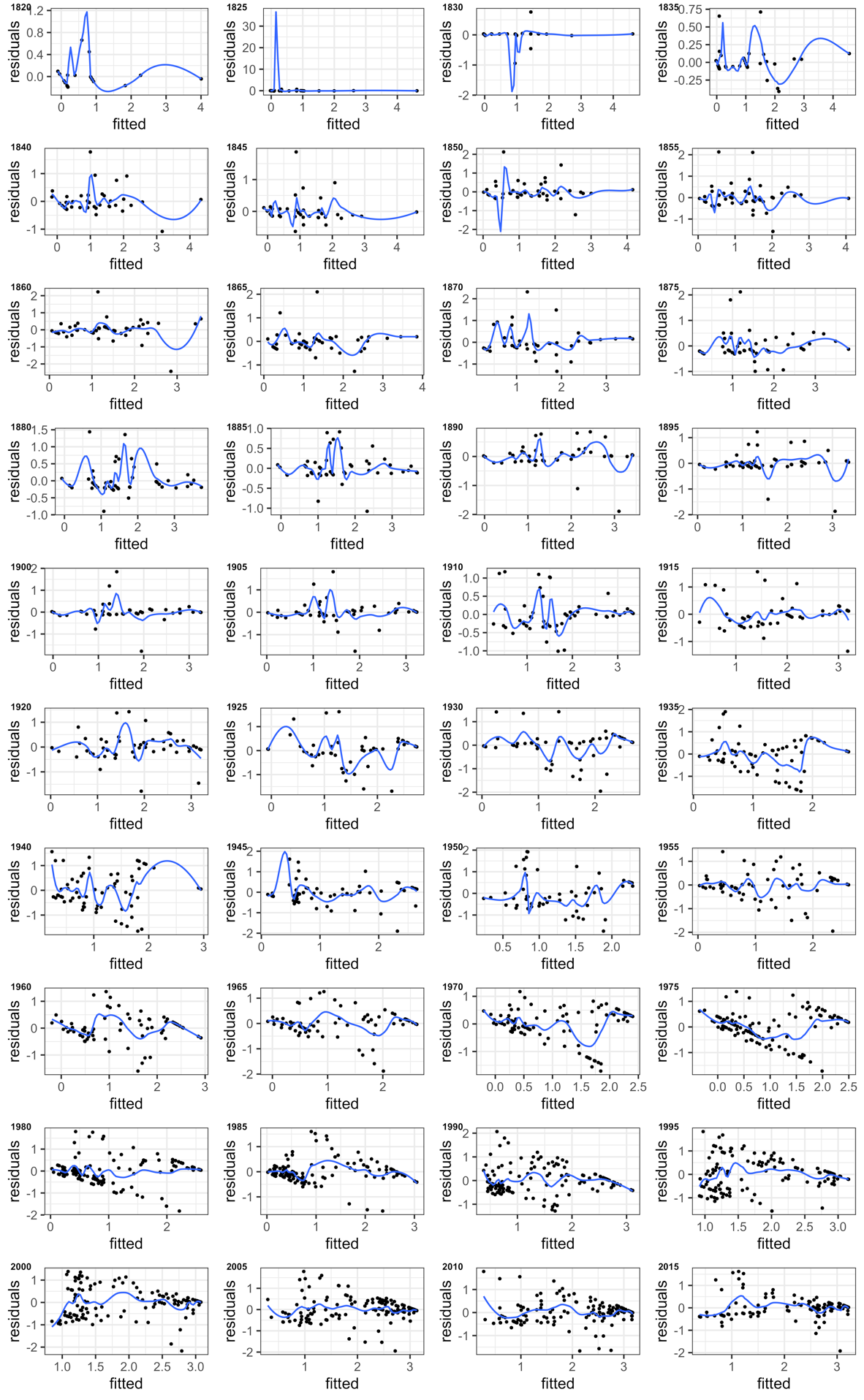


**Figure S16. Residuals against fitted values for the longitudinal analysis of Polity 5 data.** For description of the models and interpretation of residual plots see Methods; for results of these models see Fig. 4.


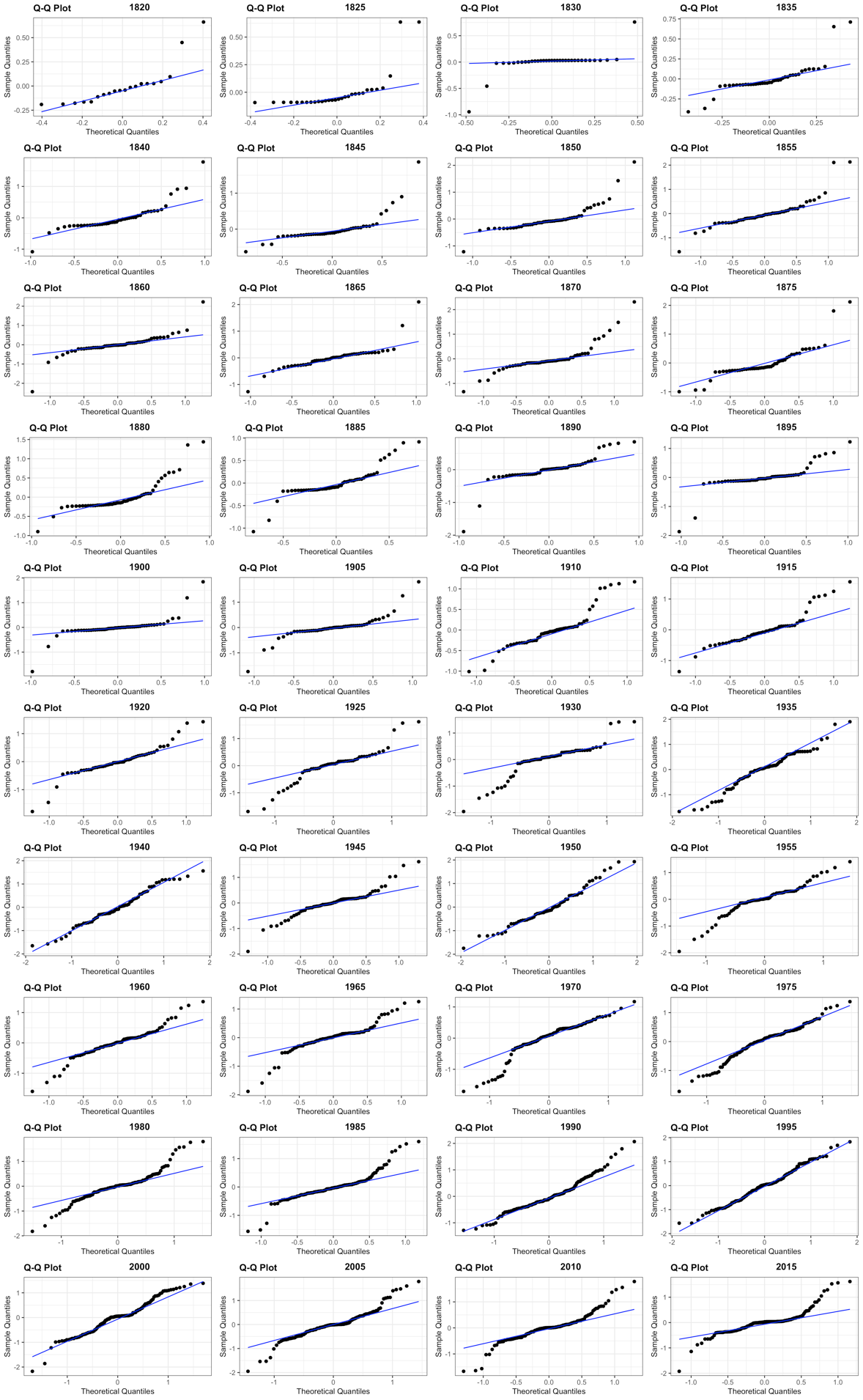


**Figure S17. Q-Q plots for the longitudinal analysis of Polity 5 data.** For description of the models and interpretation of Q-Q plots see Methods; for results of these models see Fig. 4.

**
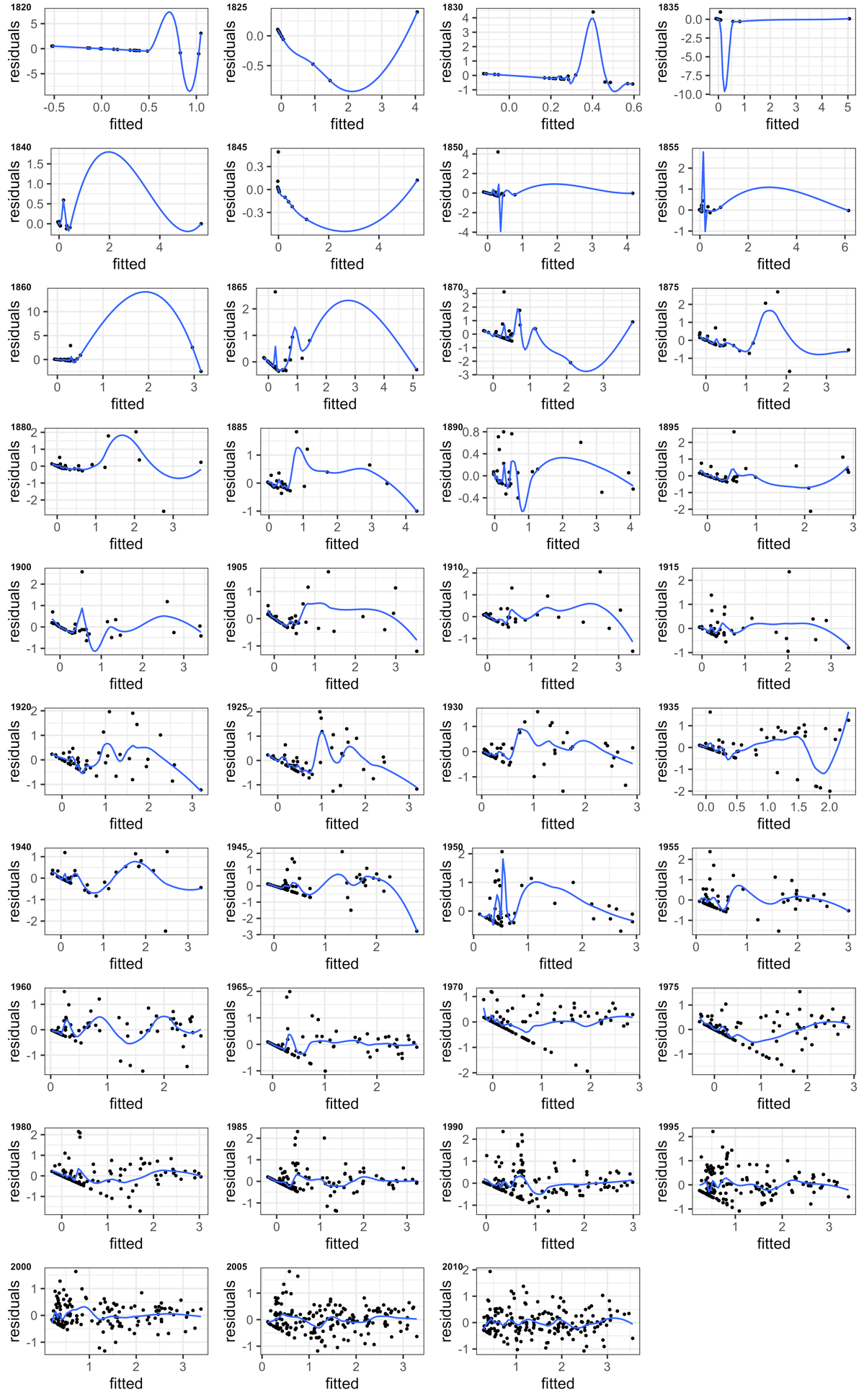
**

**Figure S18. Residuals against fitted values for the longitudinal analysis of the Vanhanen Index.** For description of the models and interpretation of residual plots see Methods; for results of these models see Fig. 4.


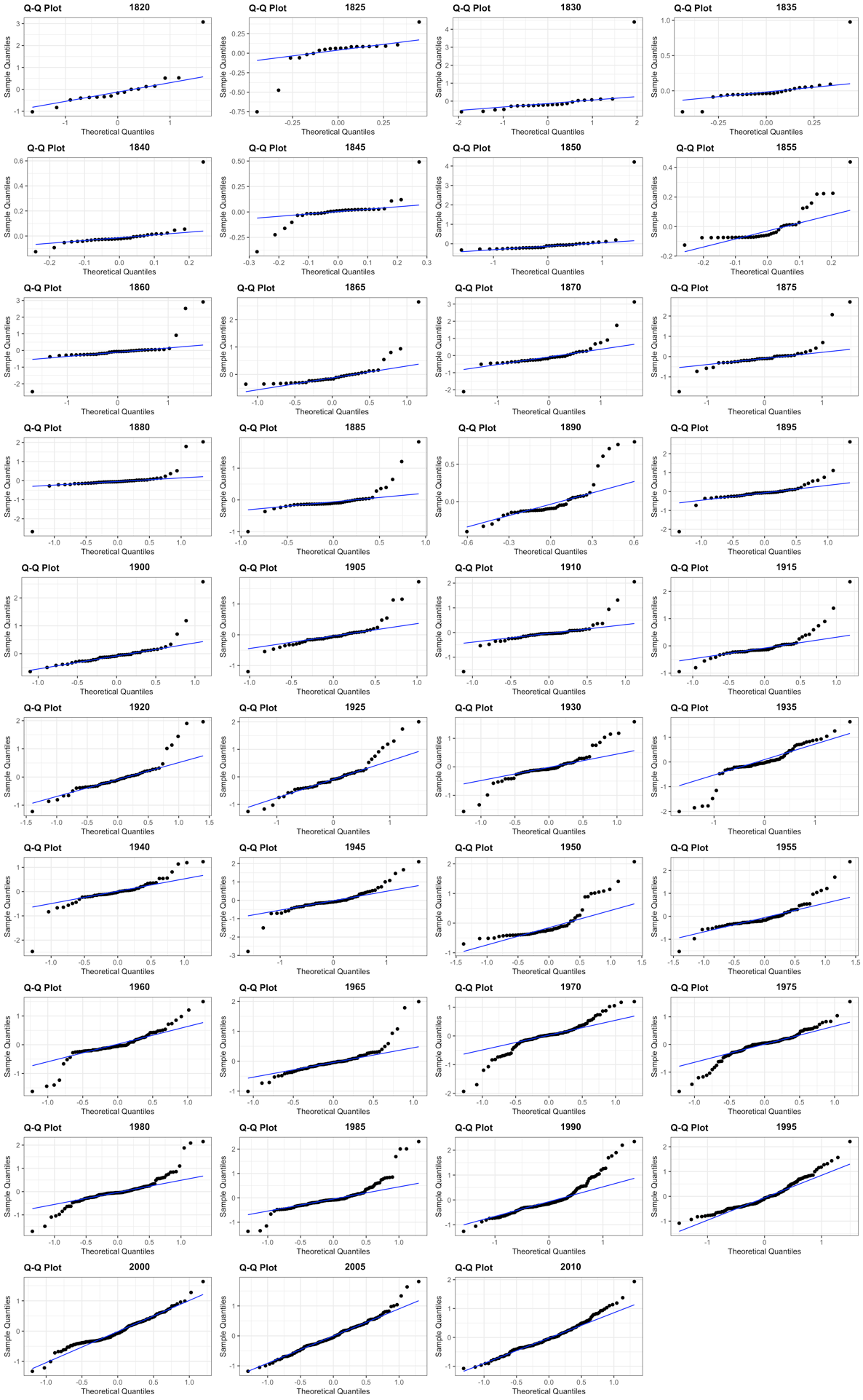


**Figure S19. Q-Q plots for the longitudinal analysis of the Vanhanen Index.** For description of the models and interpretation of Q-Q plots see Methods; for results of these models see Fig. 4.


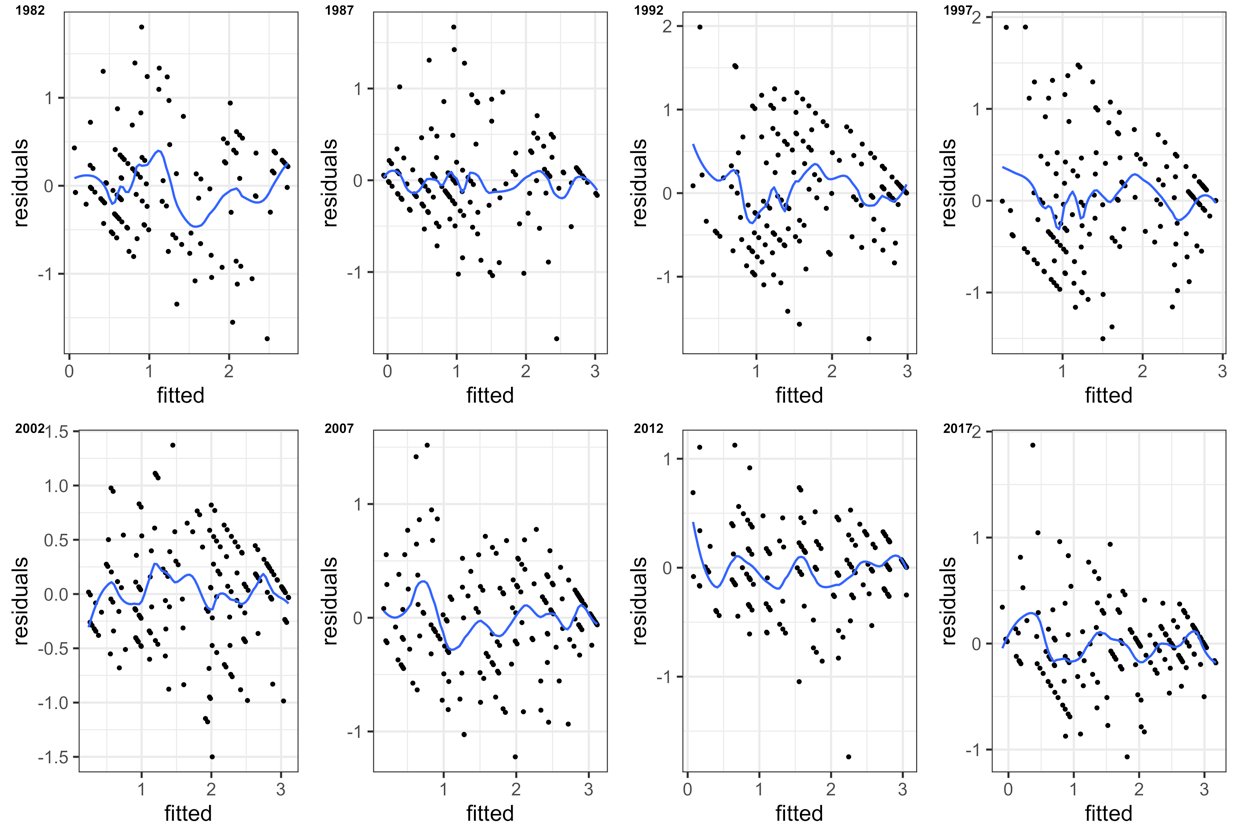


**Figure S20. Residuals against fitted values for the longitudinal analysis of Freedom House data.** For description of the models and interpretation of residual plots see Methods; for results of these models see Fig. 4.


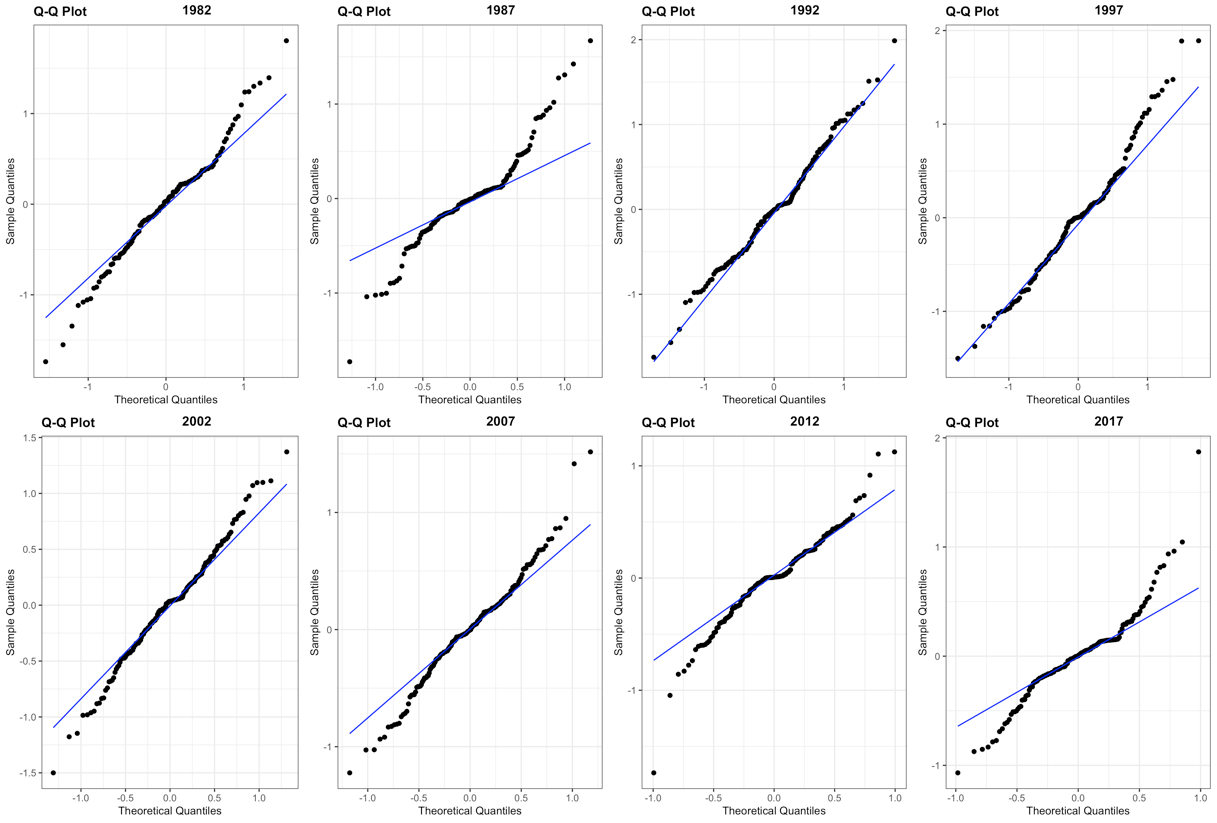


**Figure S21. Q-Q plots for the longitudinal analysis of Freedom House data.** For description of the models and interpretation of Q-Q plots see Methods; for results of these models see Fig. 4.

**Figure S22. Separate effects of geographic, linguistic, and religious connections predicting democracy.** Pairwise differences in democracy between nations were regressed on geographic, linguistic or religious connections between nations separately, at each time slice for which data was available, resulting in 1,398 cross-sectional models. Multiple regression standardized coefficients of the three predictors are presented separately for Polity 5 (**a**; 1800-2018), the Vanhanen Index (**b**; 1810-2012), and Freedom House data (**c**; 1972-2020), with 95% CI annotated. The direction and significance of effects colour-coded: red for significant positive coefficients (p < .05), pink for non-significant positive coefficients, dark blue for significant negative coefficients (p < .05), and light blue for non-significant negative coefficients. **d-f**, Semi-partial coefficients of determination (R^2^) are displayed below the respective models and outcome variables from a-c, indicating the proportion of variance in democracy explained by geography (green), language (red), or religion (blue), without controlling for the other two variables. The three waves of democratization are highlighted in gray on all graphs.

**Figure S23. Independent effects of geographic, linguistic, and religious connections in predicting democracy (using unlogged geographic proximity).** Pairwise differences in democracy between nations were simultaneously regressed on geographic, linguistic and religious connections between nations, at each time slice for which data was available, resulting in 466 cross-sectional models. Multiple regression standardized coefficients of the three predictors are presented separately for Polity 5 (**a**; 1800-2018), the Vanhanen Index (**b**; 1810-2012), and Freedom House data (**c**; 1972-2020), with 95% CI annotated. The direction and significance of effects colour-coded: red for significant positive coefficients (p < .05), pink for non-significant positive coefficients, dark blue for significant negative coefficients (p < .05), and light blue for non-significant negative coefficients. **d-f**, Semi-partial coefficients of determination (R^2^) are displayed below the respective models and outcome variables from a-c, indicating the proportion of variance in democracy explained by geography (green), language (red), or religion (blue), after controlling for the other two variables. The three waves of democratization are highlighted in gray on all graphs.

**Figure S24. Independent effects of geographic, linguistic, and religious connections predicting democracy (using alternative cultural measures).** Pairwise differences in democracy between nations were simultaneously regressed on geographic, linguistic and religious connections between nations, at each time slice for which data was available, resulting in 466 cross-sectional models. Multiple regression standardized coefficients of the three predictors are presented separately for Polity 5 (**a**; 1800-2018), the Vanhanen Index (**b**; 1810-2012), and Freedom House data (**c**; 1972-2020), with 95% CI annotated. The direction and significance of effects colour-coded: red for significant positive coefficients (p < .05), pink for non-significant positive coefficients, dark blue for significant negative coefficients (p < .05), and light blue for non-significant negative coefficients. **d-f**, Semi-partial coefficients of determination (R^2^) are displayed below the respective models and outcome variables from a-c, indicating the proportion of variance in democracy explained by geography (green), language (red), or religion (blue), after controlling for the other two variables. The three waves of democratization are highlighted in gray on all graphs.

**Figure S25.** **Independent effects of democracy among geographic, linguistic, and religious connections at T1 predicting democracy at T2 (using unlogged geographic proximity; 10-year lag).** Nations’ democracy scores at T2 were simultaneously regressed on the cumulative democracy of their geographic, linguistic and religious connections at T1 (10 years prior), after controlling for their democracy at T1. These analyses essentially trace changes in democracy over a 10-year period based on democracy in neighbouring or related nations (see also Fig. 2). Each time-slice was analysed separately for each of the three democracy measures (see Methods), resulting in 426 longitudinal models. Multiple regression standardized coefficients of the three main predictors are presented separately for Polity 5 (**a**; 1810-2018), the Vanhanen Index (**b**; 1820-2012) and Freedom House data (**c**; 1982 -2020), with 95% CI annotated. The direction and significance of effects colour-coded: red for significant positive coefficients (p < .05), pink for non-significant positive coefficients, dark blue for significant negative coefficients (p < .05), and light blue for non-significant negative coefficients. **d-f**, Semi-partial coefficients of determination (R^2^) are displayed below the respective models and outcome variables from a-c, indicating the proportion of variance in democracy explained by the cumulative democracy of geographic neighbours (green), and linguistic (red) or religious relatives (blue), after controlling for the other two variables and difference in democracy at T1. The three waves of democratization are highlighted in gray on all graphs.

**Figure S26.** **Independent effects of democracy among geographic, linguistic, and religious connections at T1 predicting democracy at T2 (using alternative cultural measures; 10-year lag).** Nations’ democracy scores at T2 were simultaneously regressed on the cumulative democracy of their geographic, linguistic and religious connections at T1 (10 years prior), after controlling for their democracy at T1. These analyses essentially trace changes in democracy over a 10-year period based on democracy in neighbouring or related nations (see also Fig. 2). Each time-slice was analysed separately for each of the three democracy measures (see Methods), resulting in 426 longitudinal models. Multiple regression standardized coefficients of the three main predictors are presented separately for Polity 5 (**a**; 1810-2018), the Vanhanen Index (**b**; 1820-2012) and Freedom House data (**c**; 1982 -2020), with 95% CI annotated. The direction and significance of effects colour-coded: red for significant positive coefficients (p < .05), pink for non-significant positive coefficients, dark blue for significant negative coefficients (p < .05), and light blue for non-significant negative coefficients. **d-f**, Semi-partial coefficients of determination (R^2^) are displayed below the respective models and outcome variables from a-c, indicating the proportion of variance in democracy explained by the cumulative democracy of geographic neighbours (green), and linguistic (red) or religious relatives (blue), after controlling for the other two variables and difference in democracy at T1. The three waves of democratization are highlighted in gray on all graphs.

**Table S1. Sampled nations and culture assignments.** List of nations sampled for this study, with information about their ISO 3166 code, capital city, and majority language and religion assignments. Capital cities were used as geographic centroids. Language data came from Ethnologue 21 (Eberhard et al., 2018) and religion data from the ARDA National Profiles (Brown et al., 2018; Finke & Grim, 2019).

| **ISO2** | **Nation** | **Capital** | **Language** | **ISO3** | **Glottocode** | **Religion** |
| --- | --- | --- | --- | --- | --- | --- |
| AD | Andorra | Andorra la Vella | Catalan | cat | stan1289 | Catholic |
| AE | United Arab Emirates | Abu Dhabi | Gulf Arabic | afb | gulf1241 | Sunni |
| AF | Afghanistan | Kabul | Southern Pashto | pbt | sout2649 | Sunni |
| AG | Antigua and Barbuda | Saint John's | English | eng | stan1293 | Protestant |
| AL | Albania | Tirana | Northern Tosk Albanian | als | tosk1239 | Sunni |
| AM | Armenia | Yerevan | Armenian | hye | nucl1235 | Orthodox |
| AO | Angola | Luanda | Portuguese | por | port1283 | Catholic |
| AR | Argentina | Buenos Aires | Spanish | spa | stan1288 | Catholic |
| AT | Austria | Vienna | Bavarian | bar | bava1246 | Catholic |
| AU | Australia | Canberra | English | eng | stan1293 | Catholic |
| AZ | Azerbaijan | Baku | North Azerbaijani | azj | nort2697 | Shia |
| BA | Bosnia and Herzegovina | Sarajevo | Bosnian | bos | bosn1245 | Sunni |
| BAD | Baden | Karlsruhe | Swiss German | gsw | swis1247 | Catholic |
| BAV | Bavaria | Munich | Bavarian | bar | bava1246 | Catholic |
| BB | Barbados | Bridgetown | English | eng | stan1293 | Protestant |
| BD | Bangladesh | Dhaka | Bengali | ben | beng1280 | Sunni |
| BE | Belgium | Brussels | Dutch | nld | dutc1256 | Catholic |
| BF | Burkina Faso | Ouagadougou | Mossi | mos | moss1236 | Sunni |
| BG | Bulgaria | Sofia | Bulgarian | bul | bulg1262 | Orthodox |
| BH | Bahrain | Manama | Baharna Arabic | abv | baha1259 | Shia |
| BI | Burundi | Bujumbura | Rundi | run | rund1242 | Catholic |
| BJ | Benin | Porto Novo | Fon | fon | fonn1241 | Sunni |
| BN | Brunei | Bandar Seri Begawan | Brunei | kxd | brun1242 | Sunni |
| BO | Bolivia | Sucre | Spanish | spa | stan1288 | Catholic |
| BR | Brazil | Brasilia | Portuguese | por | port1283 | Catholic |
| BS | Bahamas | Nassau | English | eng | stan1293 | Protestant |
| BT | Bhutan | Thimphu | Dzongkha | dzo | dzon1239 | VajrayanaBuddhism |
| BW | Botswana | Gaborone | Tswana | tsn | tswa1253 | Animism |
| BY | Belarus | Minsk | Russian | rus | russ1263 | Orthodox |
| BZ | Belize | Belmopan | English | eng | stan1293 | Catholic |
| CA | Canada | Ottawa | English | eng | stan1293 | Catholic |
| CD | Democratic Republic of the Congo | Kinshasa | Luba-Lulua | lua | luba1249 | Catholic |
| CF | Central African Republic | Bangui | Sango | sag | sang1328 | Catholic |
| CG | Congo | Brazzaville | Kituba (Congo) | mkw | kitu1245 | Catholic |
| CH | Switzerland | Bern | Swiss German | gsw | swis1247 | Catholic |
| CI | Cote d'Ivoire | Yamoussoukro | Baoule | bci | baou1238 | Sunni |
| CL | Chile | Santiago | Spanish | spa | stan1288 | Catholic |
| CM | Cameroon | Yaounde | Bulu (Cameroon) | bum | bulu1251 | Catholic |
| CN | China | Beijing | Mandarin Chinese | cmn | mand1415 | ChineseFolkReligion |
| CO | Colombia | Bogota | Spanish | spa | stan1288 | Catholic |
| CR | Costa Rica | San Jose | Spanish | spa | stan1288 | Catholic |
| CU | Cuba | Havanna | Spanish | spa | stan1288 | Catholic |
| CV | Cape Verde | Praia | Kabuverdianu | kea | kabu1256 | Catholic |
| CY | Cyprus | Nicosia | Modern Greek | ell | mode1248 | Orthodox |
| CZ | Czechia | Prague | Czech | ces | czec1258 | Catholic |
| CZE | Czechoslovakia | Prague | Czech | ces | czec1258 | Catholic |
| DE | Germany | Berlin | German | deu | stan1295 | Protestant |
| DJ | Djibouti | Jibuti | Somali | som | soma1255 | Sunni |
| DK | Denmark | Copenhagen | Danish | dan | dani1285 | Protestant |
| DM | Dominica | Roseau | Saint Lucian Creole French | acf | sain1246 | Catholic |
| DO | Dominican Republic | Santo Domingo | Spanish | spa | stan1288 | Catholic |
| DRV | Vietnam North | Hanoi | Vietnamese | vie | viet1252 | MahayanaBuddhism |
| DZ | Algeria | Algiers | Algerian Arabic | arq | alge1239 | Sunni |
| EC | Ecuador | Quito | Spanish | spa | stan1288 | Catholic |
| EE | Estonia | Tallinn | Estonian | ekk | esto1258 | Orthodox |
| EG | Egypt | Cairo | Egyptian Arabic | arz | egyp1253 | Sunni |
| ER | Eritrea | Asmara | Tigrinya | tir | tigr1271 | Sunni |
| ES | Spain | Madrid | Spanish | spa | stan1288 | Catholic |
| ET | Ethiopia | Addis Abeba | Amharic | amh | amha1245 | Orthodox |
| FI | Finland | Helsinki | Finnish | fin | finn1318 | Protestant |
| FJ | Fiji | Suva | Fiji Hindi | hif | fiji1242 | Protestant |
| FM | Micronesia | Palikir | Chuukese | chk | chuu1238 | Catholic |
| FR | France | Paris | French | fra | stan1290 | Catholic |
| GA | Gabon | Libreville | Fang (Equatorial Guinea) | fan | fang1246 | Catholic |
| GB | United Kingdom | London | English | eng | stan1293 | Anglican |
| GCL | Gran Colombia | Bogota | Spanish | spa | stan1288 | Catholic |
| GD | Grenada | Saint George's | Grenadian Creole English | gcl | gren1247 | Catholic |
| GDR | Germany East | Berlin | German | deu | stan1295 | Protestant |
| GE | Georgia | Tbilisi | Georgian | kat | nucl1302 | Orthodox |
| GFR | Germany West | Bonn | German | deu | stan1295 | Protestant |
| GH | Ghana | Accra | Akan | aka | akan1250 | Protestant |
| GM | Gambia | Banjul | Mandinka | mnk | mand1436 | Sunni |
| GMY | Prussia | Berlin | German | deu | stan1295 | Protestant |
| GN | Guinea | Conakry | Pular | fuf | pula1262 | Sunni |
| GQ | Equatorial Guinea | Malabo | Fang (Equatorial Guinea) | fan | fang1246 | Catholic |
| GR | Greece | Athens | Modern Greek | ell | mode1248 | Orthodox |
| GT | Guatemala | Guatemala | Spanish | spa | stan1288 | Catholic |
| GW | Guinea-Bissau | Bissau | Balanta-Kentohe | ble | bala1301 | Animism |
| GY | Guyana | Georgetown | Guyanese Creole English | gyn | creo1235 | Pentecostal |
| HN | Honduras | Tegucigalpa | Spanish | spa | stan1288 | Catholic |
| HR | Croatia | Zagreb | Croatian | hrv | croa1245 | Catholic |
| HT | Haiti | Port-au-Prince | Haitian | hat | hait1244 | Protestant |
| HU | Hungary | Budapest | Hungarian | hun | hung1274 | Catholic |
| ID | Indonesia | Jakarta | Javanese | jav | java1254 | Sunni |
| IE | Ireland | Dublin | English | eng | stan1293 | Catholic |
| IL | Israel | Jerusalem | Modern Hebrew | heb | hebr1245 | Judaism |
| IN | India | Ni Dilli | Hindi | hin | hind1269 | Hinduism |
| IQ | Iraq | Baghdad | Gilit Mesopotamian Arabic | acm | meso1252 | Shia |
| IR | Iran | Tehran | Western Farsi | pes | west2369 | Shia |
| IS | Iceland | Reykjavik | Icelandic | isl | icel1247 | Protestant |
| IT | Italy | Rome | Italian | ita | ital1282 | Catholic |
| JM | Jamaica | Kingston | Jamaican Creole English | jam | jama1262 | Protestant |
| JO | Jordan | 'Amman | South Levantine Arabic | ajp | sout3123 | Sunni |
| JP | Japan | Tokyo | Japanese | jpn | nucl1643 | MahayanaBuddhism |
| KE | Kenya | Nairobi | Kikuyu | kik | kiku1240 | Pentecostal |
| KG | Kyrgyzstan | Biskek | Kirghiz | kir | kirg1245 | Sunni |
| KH | Cambodia | Phnum Penh | Central Khmer | khm | cent1989 | TheravadaBuddhism |
| KI | Kiribati | Bairiki | Gilbertese | gil | gilb1244 | Catholic |
| KM | Comoros | Moroni | Ngazidja Comorian | zdj | ngaz1238 | Sunni |
| KN | Saint Kitts and Nevis | Basseterre | Antigua and Barbuda Creole English | aig | anti1245 | Protestant |
| KOR | Korea | Seoul | Korean | kor | kore1280 | Confucianism |
| KP | North Korea | Pyongyang | Korean | kor | kore1280 | MahayanaBuddhism |
| KR | South Korea | Seoul | Korean | kor | kore1280 | MahayanaBuddhism |
| KW | Kuwait | al-Kuwayt | Gulf Arabic | afb | gulf1241 | Sunni |
| KZ | Kazakhstan | Astana | Kazakh | kaz | kaza1248 | Sunni |
| LA | Laos | Vientiane | Lao | lao | laoo1244 | TheravadaBuddhism |
| LB | Lebanon | Bayrut | North Levantine Arabic | apc | nort3139 | Sunni |
| LC | Saint Lucia | Castries | Saint Lucian Creole French | acf | sain1246 | Catholic |
| LI | Liechtenstein | Vaduz | Swiss German | gsw | swis1247 | Catholic |
| LK | Sri Lanka | Colombo | Sinhala | sin | sinh1246 | TheravadaBuddhism |
| LR | Liberia | Monrovia | Liberia Kpelle | xpe | libe1247 | Pentecostal |
| LS | Lesotho | Maseru | Southern Sotho | sot | sout2807 | Catholic |
| LT | Lithuania | Vilnius | Lithuanian | lit | lith1251 | Catholic |
| LU | Luxembourg | Luxemburg | Luxembourgish | ltz | luxe1241 | Catholic |
| LV | Latvia | Riga | Latvian | lav | latv1249 | Protestant |
| LY | Libya | Tripoli | Libyan Arabic | ayl | liby1240 | Sunni |
| MA | Morocco | Rabat | Moroccan Arabic | ary | moro1292 | Sunni |
| MC | Monaco | Monaco-Ville | French | fra | stan1290 | Catholic |
| MD | Moldova | Chisinau | Romanian | ron | roma1327 | Orthodox |
| ME | Montenegro | Podgorica | Bosnian | bos | bosn1245 | Orthodox |
| MG | Madagascar | Antananarivo | Plateau Malagasy | plt | plat1254 | Animism |
| MH | Marshall Islands | Rita | Marshallese | mah | mars1254 | Protestant |
| MK | Macedonia | Skopje | Macedonian | mkd | mace1250 | Orthodox |
| ML | Mali | Bamako | Bambara | bam | bamb1269 | Sunni |
| MM | Myanmar | Rangoon | Burmese | mya | nucl1310 | TheravadaBuddhism |
| MN | Mongolia | Ulaanbaatar | Halh Mongolian | khk | halh1238 | VajrayanaBuddhism |
| MOD | Modena | Modena | Italian | ita | ital1282 | Catholic |
| MR | Mauritania | Nouakchott | Hassaniyya | mey | hass1238 | Sunni |
| MT | Malta | Valletta | Maltese | mlt | malt1254 | Catholic |
| MU | Mauritius | Port Louis | Morisyen | mfe | mori1278 | Catholic |
| MV | Maldives | Male | Dhivehi | div | dhiv1236 | Sunni |
| MW | Malawi | Lilongwe | Nyanja | nya | nyan1308 | Protestant |
| MX | Mexico | Mexico City | Spanish | spa | stan1288 | Catholic |
| MY | Malaysia | Kuala Lumpur | Colloquial Malay | zlm | mala1479 | Sunni |
| MZ | Mozambique | Maputo | Makhuwa | vmw | makh1264 | Catholic |
| NA | Namibia | Windhoek | Kuanyama | kua | kuan1247 | Protestant |
| NE | Niger | Niamey | Hausa | hau | haus1257 | Sunni |
| NG | Nigeria | Abuja | Yoruba | yor | yoru1245 | Sunni |
| NI | Nicaragua | Managua | Spanish | spa | stan1288 | Catholic |
| NL | Netherlands | Amsterdam | Dutch | nld | dutc1256 | Catholic |
| NO | Norway | Oslo | Norwegian | nor | norw1258 | Protestant |
| NP | Nepal | Kathmandu | Nepali | npi | nepa1254 | Sunni |
| NR | Nauru | Yaren | Nauru | nau | naur1243 | Protestant |
| NZ | New Zealand | Wellington | English | eng | stan1293 | Protestant |
| OFS | Orange Free State | Bloemfontein | Southern Sotho | sot | sout2807 | Protestant |
| OM | Oman | Muscat | Omani Arabic | acx | oman1239 | Ibadiyya |
| PA | Panama | Panama | Spanish | spa | stan1288 | Catholic |
| PAP | Papal States | Rome | Italian | ita | ital1282 | Catholic |
| PE | Peru | Lima | Spanish | spa | stan1288 | Catholic |
| PG | Papua New Guinea | Port Moresby | Enga | enq | enga1252 | Protestant |
| PH | Philippines | Manila | Tagalog | tgl | taga1270 | Catholic |
| PK | Pakistan | Islamabad | Western Panjabi | pnb | west2386 | Sunni |
| PL | Poland | Warsaw | Polish | pol | poli1260 | Catholic |
| PMA | Parma | Parma | Italian | ita | ital1282 | Catholic |
| PT | Portugal | Lisbon | Portuguese | por | port1283 | Catholic |
| PW | Palau | Koror | Palauan | pau | pala1344 | Catholic |
| PY | Paraguay | Asuncion | Paraguayan Guarani | gug | para1311 | Catholic |
| QA | Qatar | Doha | Gulf Arabic | afb | gulf1241 | Sunni |
| RO | Romania | Bucharest | Romanian | ron | roma1327 | Orthodox |
| RS | Serbia | Belgrade | Serbian | srp | serb1264 | Orthodox |
| RU | Russia | Moscow | Russian | rus | russ1263 | Orthodox |
| RVN | Vietnam South | Saigon | Vietnamese | vie | viet1252 | MahayanaBuddhism |
| RW | Rwanda | Kigali | Kinyarwanda | kin | kiny1244 | Catholic |
| SA | Saudi Arabia | Riyadh | Hijazi Arabic | acw | hija1235 | Sunni |
| SAR | Sardinia | Turin | Italian | ita | ital1282 | Catholic |
| SAX | Saxony | Dresden | Upper Saxon | sxu | uppe1400 | Protestant |
| SB | Solomon Islands | Honiara | Kwara'ae | kwf | kwar1239 | Protestant |
| SC | Seychelles | Victoria | Seselwa Creole French | crs | sese1246 | Catholic |
| SD | Sudan | Khartoum | Sudanese Arabic | apd | suda1236 | Sunni |
| SE | Sweden | Stockholm | Swedish | swe | swed1254 | Protestant |
| SG | Singapore | Singapore | Mandarin Chinese | cmn | mand1415 | MahayanaBuddhism |
| SI | Slovenia | Ljubljana | Slovenian | slv | slov1268 | Catholic |
| SIC | Two Sicilies | Naples | Continental Southern Italian | nap | neap1235 | Catholic |
| SK | Slovakia | Bratislava | Slovak | slk | slov1269 | Catholic |
| SL | Sierra Leone | Freetown | Mende (Sierra Leone) | men | mend1266 | Sunni |
| SM | San Marino | San Marino | Italian | ita | ital1282 | Catholic |
| SN | Senegal | Dakar | Wolof | wol | nucl1347 | Sunni |
| SO | Somalia | Mogadishu | Somali | som | soma1255 | Sunni |
| SR | Suriname | Paramaribo | Dutch | nld | dutc1256 | Hinduism |
| SS | South Sudan | Juba | Nuer | nus | nuer1246 | Catholic |
| ST | Sao Tome and Principe | Sao Tome | Saotomense | cri | saot1239 | Catholic |
| SUD | Sudan | Khartoum | Sudanese Arabic | apd | suda1236 | Sunni |
| SV | El Salvador | San Salvador | Spanish | spa | stan1288 | Catholic |
| SY | Syria | Damascus | North Levantine Arabic | apc | nort3139 | Sunni |
| SZ | Swaziland | Mbabane | Swati | ssw | swat1243 | Pentecostal |
| TD | Chad | N'Djamena | Chadian Arabic | shu | chad1249 | Sunni |
| TG | Togo | Lome | Kabiye | kbp | kabi1261 | Animism |
| TH | Thailand | Bangkok | Thai | tha | thai1261 | TheravadaBuddhism |
| TJ | Tajikistan | Dushanbe | Tajik | tgk | taji1245 | Sunni |
| TL | Timor-Leste | Dili | Tetun Dili | tdt | tetu1246 | Catholic |
| TM | Turkmenistan | Asgabat | Turkmen | tuk | turk1304 | Sunni |
| TN | Tunisia | Tunis | Tunisian Arabic | aeb | tuni1259 | Sunni |
| TO | Tonga | Nuku'alofa | Tonga (Tonga Islands) | ton | tong1325 | Protestant |
| TR | Turkey | Ankara | Turkish | tur | nucl1301 | Sunni |
| TT | Trinidad and Tobago | Port of Spain | English | eng | stan1293 | Protestant |
| TUS | Tuscany | Florence | Italian | ita | ital1282 | Catholic |
| TV | Tuvalu | Vaiaku | Tuvalu | tvl | tuva1244 | Protestant |
| TW | Taiwan | Taipei | Min Nan Chinese | nan | minn1241 | Protestant |
| TZ | Tanzania | Dodoma | Swahili | swh | swah1253 | Catholic |
| UA | Ukraine | Kiev | Ukrainian | ukr | ukra1253 | Orthodox |
| UG | Uganda | Kampala | Ganda | lug | gand1255 | Catholic |
| UPC | United Province CA | Quebec City | English | eng | stan1293 | Catholic |
| US | United States | Washington | English | eng | stan1293 | Protestant |
| USR | USSR | Moscow | Russian | rus | russ1263 | Orthodox |
| UY | Uruguay | Montevideo | Spanish | spa | stan1288 | Catholic |
| UZ | Uzbekistan | Tashkent | Northern Uzbek | uzn | nort2690 | Sunni |
| VC | Saint Vincent and the Grenadines | Kingstown | Vincentian Creole English | svc | vinc1243 | Protestant |
| VE | Venezuela | Caracas | Spanish | spa | stan1288 | Catholic |
| VN | Vietnam | Ha Noi | Vietnamese | vie | viet1252 | Animism |
| VU | Vanuatu | Vila | Lenakel | tnl | lena1238 | Protestant |
| WRT | Wuerttemburg | Stuttgart | Swabian | swg | swab1242 | Protestant |
| WS | Samoa | Apia | Samoan | smo | samo1305 | Protestant |
| XK | Kosovo | Pristina | Gheg Albanian | aln | gheg1238 | Sunni |
| YAR | Yemen North | Sana'a | Sanaani Arabic | ayn | sana1295 | Shia |
| YE | Yemen | San'a | Sanaani Arabic | ayn | sana1295 | Shia |
| YGS | Serbia and Montenegro | Belgrade | Serbian | srp | serb1264 | Orthodox |
| YPR | Yemen South | Aden | Ta'izzi-Adeni Arabic | acq | taiz1242 | Sunni |
| YUG | Yugoslavia | Belgrade | Serbian | srp | serb1264 | Orthodox |
| ZA | South Africa | Pretoria | Zulu | zul | zulu1248 | Protestant |
| ZM | Zambia | Lusaka | Bemba (Zambia) | bem | bemb1257 | Protestant |
| ZW | Zimbabwe | Harare | Shona | sna | shon1251 | Protestant |

**Table S2. Correlations between predictors.** Pearson’s r correlation between lower triangles of predictor matrices: geographic proximity (geoprox), alternative geographic proximity (geoprox.alt), geographic contiguity (geo.cont), linguistic connection (lingcon), alternative linguistic connection (lingcon.alt), linguistic contiguity (ling.cont), religious connection (relcon), alternative religious connection (relcon.alt), and religious contiguity (rel.cont). See Methods for more details on how each network was produced. All correlations are significant at a level of *p* < .001.

|  | **geoprox.alt** | **geo.cont** | **lingcon** | **lingcon.alt** | **ling.cont** | **relcon** | **relcon.alt** | **rel.cont** |
| --- | --- | --- | --- | --- | --- | --- | --- | --- |
| **geoprox** | .901 | .357 | .295 | .355 | .114 | .173 | .181 | .126 |
| **geoprox.alt** |  | .197 | .217 | .257 | .083 | .113 | .119 | .092 |
| **geo.cont** |  |  | .125 | .254 | .103 | .063 | .082 | .105 |
| **lingcon** |  |  |  | .615 | .191 | .388 | .392 | .186 |
| **lingcon.alt** |  |  |  |  | .525 | .327 | .356 | .312 |
| **ling.cont** |  |  |  |  |  | .141 | .153 | .168 |
| **relcon** |  |  |  |  |  |  | .982 | .524 |
| **relcon.alt** |  |  |  |  |  |  |  | .596 |

**Supplementary Methods**

**Justifications for Time-Dated Religion Tree Structure** (see Supplementary References for a list of references)

1. Protestantism was used in this analysis because it is consistently available from the religion demographic sources across most countries. However, Protestantism is a “paraphyletic” group because it includes Lutherans, Presbyterians, Baptists, and Methodists. Lutherans and Presbyterians date their split from Catholicism to Martin Luther’s theses of 1520 AD, while Baptists and Methodists are derived from the Anglican tradition over a century later. Anglicanism split with the Roman church in 1532. Since the 1520 and 1532 are so close together, and because of the paraphyly, we made a polytomy for these three taxa (Protestant, Anglican, Catholic) at 1520 (Carson 2003; Cross and Livingstone 2005; Hillerbrand 2005; Melton 2009).
2. Substantial debate exists about when Judaism and Christianity became fully distinct. We resolved this by picking a midpoint between the earliest date argued for – which is the destruction of the second Jewish Temple in 70AD, and the latest plausible date, which is the Edict of Milan by Emperor Constantine in 313 that gave Christianity legal status in the Roman empire (note, it did not at that time make Christianity the state religion, nor did it ban the practice of traditional Roman paganism). The midpoint of 70 and 313 is 192AD, which is comfortably close to the date many scholars put for the Jewish-Christian split (Cohen 2013).
3. Another unclear date is the origin of the Nation of Islam lineage. The earliest date would be ~1900 with the start of the Moorish Science Temple, but at that time the religion had little to do with orthodox Islam. More orthodox Muslim beliefs were only solidified within Nation of Islam in the 1980s. We used the midpoint of 1940 (Melton and Baumann 2010).
4. The Alawite split is dated to 873 when it’s founder Ibn Nusayr was excommunicated (Freidman 2010).
5. There are three taxa in particular for whom some scholars argue a hybrid origin among other taxa in this tree. Those would be the Alawites (hybrid Shi’a and Orthodox) and Sikhs (hybrid Hindu and Sufi Islam – which would be Sunni in our tree because Sufi is not a separate taxon), and the basal branch for Islam (hybrid Judaism and Christianity) (Melton 2010).
6. Another midpoint date is 1000 BC for the basal branch that leads to Hinduism, includes Zoroastrian, but excludes East Asian traditions. We made this decision based on a consensus in the literature that the Abrahamic, Zoroastrian, and Vedic religions all share a common ancestral belief system that circulated from Mesopotamia to India (Boyce 1979; Boyce 1984; Kak 2003; Levitt 2003). We dated this split based on 1000 BC being the midpoint of estimates for the initial composition for a mostly *complete* Vedic corpus, which are the foundational scripture that sets apart Hinduism from other traditions (Levitt 2003). The earliest Veda, the Rgveda, contains no mention of distinctive features of Hinduism such as caste and sati, and thus does not provide a date for the differentiation of Hindu thought as distinct from other Indo-Iranian religious systems (Jamison and Brereton 2014).
7. However, we estimated the split of the tradition leading to Hinduism from the East Asian group at 1500 BC– which is the generally accepted date for the composition of the earliest text among the Vedas (Rgveda). Again, while this text does not differentiate Hinduism, it does mark out distinct Indo-Mesopotamian ideas (e.g. personal yet transcendent gods, extensive debate about the correct gods for worship, specific and non-replicable divine revelations) that differentiate this tradition from the East Asian ones (Levitt 2003).
8. Jains themselves claim their religion predates Buddhism, but all scholars accept documentary evidence that the Buddha and the founder of Jainism were contemporaries.
9. Pentecostals originally came out of a subset of the Methodist movement called the Holiness movement. They are dated in the tree to 1901, when one of their founders, Agnes Ozman, experienced speaking in tongues. Methodists are included within the “Protestant” taxon in the tree, so Pentecostal is a split off Protestant (BBC 2009).
10. Vajrayana is sometimes seen as affiliated with Mahayana and sometimes seen as a wholly separate tradition of Buddhism. Although there is geographic proximity with Mahayana, Vajrayana historically appears most attributed to a separate and later influx of Buddhism into Tibet from India sometime around 900 AD +- ~100 years. This influx is associated with the teacher Padmasambhava and the most solid date is from fragments of a manuscript called the Testament of Ba. It becomes a polytomy in our tree because this all represents a separate influx from India after the diffusion of Theravada and Mahayana to the South and North respectively. Because Buddhism in India died out in the 12^th^ century, there is no Indian sister taxon to Vajrayana – if there were it would date to ~900AD (van Schaik and Iwao 2008).
11. Chinese Folk Religion is associated with the Han ethnic group. We have dated it to the rise and spread of the Han group in 1000 BC (Kim 2010; Wong 2011).
12. The early development of Confucianism as more of a religion than a political philosophy occurred shortly after Confucius’ death and drew on Taoism as a substrate. Taoism and Confucianism emerge at about the same time in China, and there is some early interaction among them (Duignan n.d.; Lumen Learning n.d. Cartwright 2017).
13. We have placed Shinto as a polytomy with Confucianism and Taoism, although recognizing that Shinto origins are unclear. We did not add it to a series of different positions in a tree block, as we did for hybrid religions, partly to maintain that our tree block reflects averaging over hybridization rather than phylogenetic uncertainty. By putting it in a polytomy, however, we effectively placed it at the midpoint of various speculative positions proposed for Shinto. Additionally, the placement of Shinto has less impact on our comparative analysis because it is a religious isolate in that it is present only in Japan to any significant extent (Cartwright 2017; Hirai 2020; Jinam 2012; Kim 2010; Matsumoto 2009; Webzine 2019).
14. We separated animism into American, Oceanic, African and Eurasian branches, which we placed as a polytomy of outgroups to the major world religions, reflecting their independent trajectories around the globe. The timing of the breakup of these lineages is unknown and could stretch back to the human expansion from Africa more than 50kya. However, this would produce a religious distance matrix dominated by distances between these traditions, with little relative difference in the breakup of the world religions that represent most of the global population. To avoid this loss of information, we simply assigned an overall tree depth of 4000 years, 500 years older than any other lineages in the tree

**Supplementary References**

ARDA. *Family Trees for American Religion*, <<https://www.thearda.com/denoms/families/trees/index.asp>> (n.d.).

Bahá'ís of the United States. *Origins of the Bahá'í Faith*, <<https://www.bahai.us/beliefs/origins/>> (n.d.).

BBC. *Introduction: Pentacostalism*, <<https://www.bbc.co.uk/religion/religions/christianity/subdivisions/pentecostal_1.shtml>> (2009).

Boyce, M. *Zoroastrians: Their Religious Beliefs and Practices*. (Routledge, 1979).

Boyce, M. Zoroastrianism. In: *A Handbook of Living Religions*. Ed. J. R. Hinnells. (Penguin Books, 1984).

Carson, T. *New Catholic Encyclopedia*. Vol. 2nd edition (Gale Research Inc, 2003).

Cartwright, M. Shinto. *World History Encyclopedia* (2017).

Cohen, S. J. The ways that parted: Jews, Christians, and Jewish-Christians ca. 100-150 CE. *Harvard University Press* (2013).

Cross, F. L. & Livingstone, E. A., (Eds.). *The Oxford Dictionary of the Christian Church*. (Oxford University Press, 2005).

Duignan, B. What is the Difference Between Daoism and Confucianism? *Britannica* (n.d.).

Freidman, Y. *The Nuṣayrī-ʿAlawīs: An Introduction to the Religion, History and Identity of the Leading Minority in Syria*. 8 (Brill Academic Publishers, 2010).

Hillerbrand, H. J. E. *The Oxford Encyclopedia of the Reformation*. (Oxford University Press, 2005).

Hirai, N. Shintō. *Encyclopedia Britannica* (2020).

Jamison, S. and J. Brereton. *The Rigveda: The Earliest Religious Poetry of India*. (Oxford University Press, 2014, pp. 50-59).

Kak, S. Vedic elements in the ancient Iranian religion of Zarathushtra. *The Adyar Library Bulletin*. **67**, 47-63, (2003).

Kim, C. The Concept of "Korean Religion" and Religious Studies in Korea. *Journal of Korean Religions* **1**, 23-41 (2010).

Jinam, T. *et al.* The history of human populations in the Japanese Archipelago inferred from genome-wide SNP data with a special reference to the Ainu and the Ryukyuan populations. *Journal of Human Genetics* **57**, 787-795, doi:10.1038/jhg.2012.114 (2012).

Levitt, S.H. The dating of the Indian tradition. *Anthropos*. **98**, 341-359, (2003)

Lumen Learning. *Taoism and Confucianism*, <<https://courses.lumenlearning.com/wm-introductiontosociology/chapter/taoism-and-confucianism/>> (n.d.).

Matsumoto, H. The origin of the Japanese race based on genetic markers of immunoglobulin G. *Proceedings of the Japan Academy, Series B, Physical and Biological Sciences* **85**, 69-82, doi:10.2183/pjab.85.69 (2009).

Melton, J. G. *Melton's Encyclopedia of American Religion*. Vol. 8th edition (Gale, 2009).

Melton, J. G. & Baumann, M. *Religions of the World: A Comprehensive Encylopedia of Beliefs and Practices*. Vol. 2nd edition (2010).

Synan, V. The Rise of Pentecostalism: Christian History Timeline. *Christianity Today* (n.d.).

U.S. History. *Taoism and Confucianism — Ancient Philosophies*, <<https://www.ushistory.org/civ/9e.asp>> (n.d).

van Schaik, S. & Iwao, K. Fragments of the "Testament of Ba" from Dunhuang. *Journal of the American Oriental Society* **128**, 477-487 (2008).

Webzine, U. Ainu Rights In Japan: Is Recognition Enough? *The Prespective* (2019).

Wong, W. Y. Defining Chinese Folk Religion: A Methodological Interpretation. *Asian Philosophy* **21**, 153-170, doi:10.1080/09552367.2011.563993 (2011).
